# A longitudinal investigation of risk perceptions and adaptation behavior in the US Gulf Coast

**DOI:** 10.1093/pnasnexus/pgae099

**Published:** 2024-04-09

**Authors:** Gabrielle Wong-Parodi, Daniel P Relihan, Dana Rose Garfin

**Affiliations:** Department of Earth System Science, Stanford University, Stanford, CA 94305, USA; Woods Institute for the Environment, Stanford University, Stanford, CA 94305, USA; Department of Environmental Social Sciences, Stanford University, Stanford, CA 94305, USA; Department of Psychological Science, University of California at Irvine, Irvine, CA 92697, USA; Department of Community Health Sciences, Fielding School of Public Health, University of California at Los Angeles, Los Angeles, CA 90095, USA

**Keywords:** climate change, adaptation behavior, tropical cyclones, negative experience, model

## Abstract

Climate change is occurring more rapidly than expected, requiring that people quickly and continually adapt to reduce human suffering. The reality is that climate change-related threats are unpredictable; thus, adaptive behavior must be continually performed even when threat saliency decreases (e.g. time has passed since climate-hazard exposure). Climate change-related threats are also intensifying; thus, new or more adaptive behaviors must be performed over time. Given the need to sustain climate change-related adaptation even when threat saliency decreases, it becomes essential to better understand how the relationship between risk perceptions and adaptation co-evolve over time. In this study, we present results from a probability-based representative sample of 2,774 Texas and Florida residents prospectively surveyed 5 times (2017–2022) in the presence and absence of exposure to tropical cyclones, a climate change-related threat. Distinct trajectories of personal risk perceptions emerged, with higher and more variable risk perceptions among the less educated and those living in Florida. Importantly, as tropical cyclone adaptation behaviors increased, personal risk perceptions decreased over time, particularly in the absence of storms, while future tropical cyclone risk perceptions remained constant. In sum, adapting occurs in response to current risk but may inhibit future action despite increasing future tropical cyclone risks. Our results suggest that programs and policies encouraging proactive adaptation investment may be warranted.

**Significance Statement**

Climate change-related threats are unpredictable; thus, adaptive behavior must be continuously performed even when threat saliency decreases. Given the need to sustain climate change-related adaptation even when threat saliency decreases, it becomes essential to better understand how the relationship between risk perceptions and adaptation co-evolve over time. In this study, we present results from a representative sample of 2,774 Texas and Florida residents surveyed 5 times (2017–2022) in the presence and absence of tropical cyclones. We find that adapting addresses current risk but may inhibit future action despite increasing future tropical cyclone risks. Our results suggest that programs and policies encouraging proactive adaptation investment may be warranted.

## Introduction

Climate change is occurring more rapidly than expected (1) and requires that people quickly and continually adapt (2) to reduce human suffering. The reality of climate change-related threats like tropical cyclones, wildfires, or droughts is that they are stochastic (3). Therefore, adaptive behavior must be performed continually, even when threat saliency decreases. Climate change-related threats are also intensifying (3); thus, new or more adaptive behaviors must be performed over time. Cognitions including risk perceptions are positively correlated with adaptation (4), yet performing adaptation (e.g. household preparedness) may dampen risk perceptions and inhibit future adaptation (5–8) as impacts increase. In other words, people may perceive a decrease in threat after they have undertaken efforts to mitigate its consequences. Given the need to sustain climate change-related adaptation even when threat saliency decreases, understanding the co-evolving relationship between risk perceptions and adaptation over time may help inform the design of behavioral interventions that promote effective adaptation. In this study, we ask: Does adapting today negatively correlate with climate change-related threat risk perceptions, and subsequently correlate with less adaptation in the future? Or do people continue to adapt, even in the absence of an immediate threat?

Approximately 179 million people experience tropical cyclones every year (9, 10), with dire health and economic impacts (11–13). Although the number of tropical cyclones has declined over the past 30 years (14), the levels of damage have risen due to increased storm intensity (15) caused by climate change and human development in coastal areas. Recent estimates suggest that tropical cyclones will increasingly intensify this century across many regions of the world (16), threatening the habitability of coastal communities that millions of people rely upon for their health, livelihoods, and intergenerational sustainability (17). The increasing intensity of tropical cyclones, combined with concurrent hazards and growing coastal populations, creates a dangerous situation that turns the annual tropical cyclone season into compounding and cascading disasters globally (18). Urgent action is necessary to mitigate risks and prevent further devastating consequences (19–22), especially for those for whom migration away from at-risk coastal areas is not an option due to economic burden or is not desired (17). Adaptation for tropical cyclones, for example, household preparedness (4, 23), reduces economic damage (24, 25), adverse health outcomes (26), and psychological distress (26). Effective adaptation, like household preparedness, can increase community and societal resilience (22), but it must be sustained.

While adaptation like purchasing insurance reduces impacts (27) and, in aggregate, increases collective resilience, low rates of adaptation remain stubbornly persistent (28). Low adaptation to increasing risks from climate change-related threats like tropical cyclones has exacerbated deleterious impacts (22). Studies investigating the “adaptation gap” are largely cross-sectional and explore relationships such as cost, access, awareness, sense of efficacy, and trust in authorities and experts with behaviors (4, 28, 29).

A recent meta-analysis (4) found that risk perceptions and adaptation were positively correlated (*r* = 0.20) when examined cross-sectionally, with effect sizes ranging from *r* = −0.18 to *r* = 0.60. Generally, experiences with extreme weather events (30–34), such as negative experiences from tropical cyclones (35, 36), positively correlate with risk perceptions, although exceptions exist (37). Hence, negative experiences with climate change-related threats like tropical cyclones may increase risk perceptions. Studies have also found a null or negative association between risk perceptions and adaptation (29), perhaps due to factors like misaligned motivations, perceptions of responsibility for protection, lack of trust, or inability to effect change, among other social, resource, or contextual factors (29, 38–40). Other emerging evidence suggest a more complex relationship between risk perceptions and adaptation (5–8), whereby adapting may depress risk perceptions.

**Fig. 1.**
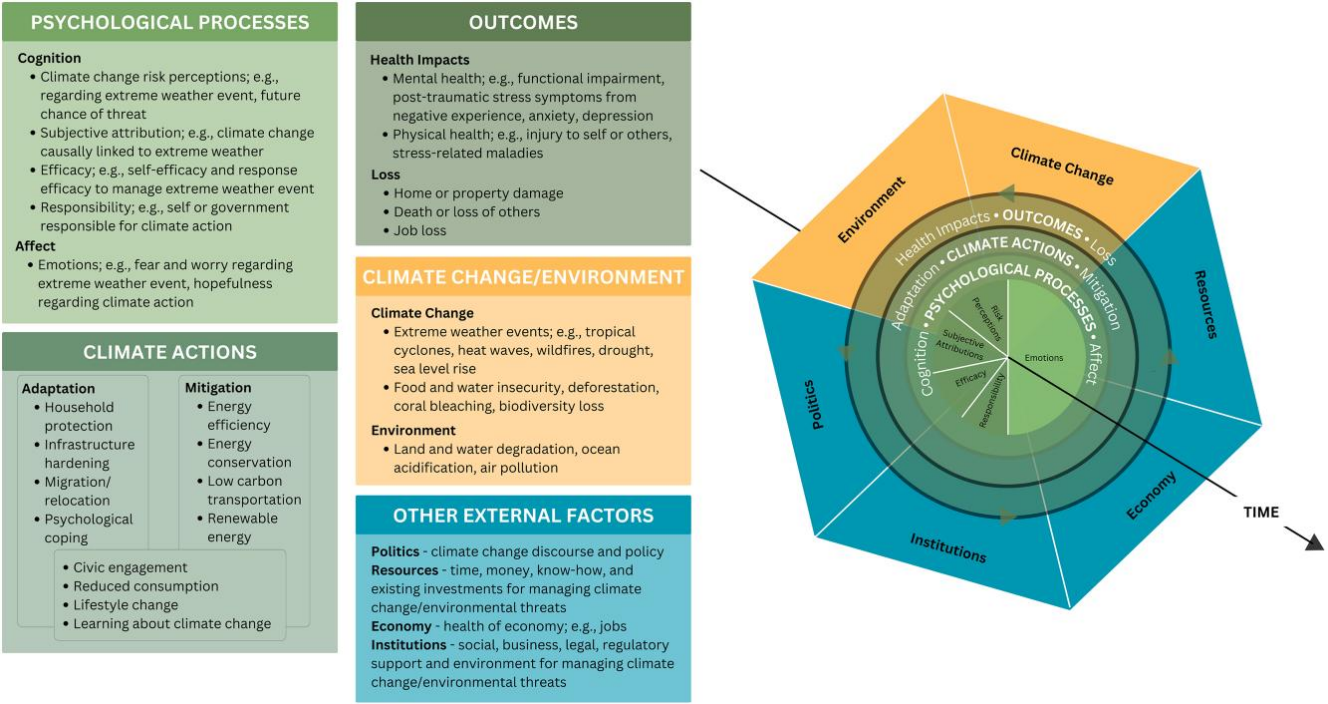
Dynamic Model of Climate Action. This conceptual model shows the proposed dynamic and reciprocal relationships among psychological processes (i.e. risk perceptions), climate action (i.e. adaptation), and outcomes under increasing climate change threats and exposures, including other external factors. DMCA is inspired and adapted from the Model of Private Proactive Adaptation to Climate Change (44), Protective Action Decision Model (42), Framework of Behavioral Adaptation to Climate Change (45), and Carman and Zint's (23) conceptualization of adaptation behaviors.

Most studies examining risk perceptions (41–43) and adaptation have been cross-sectional or theoretical, limiting assessment of causality, reciprocal, or co-evolving relationships. In a rare longitudinal study on the topic conducted over several years, Wheeler et al. (6) found that Australian farmers (*n* = 275) with higher climate change risk perceptions in 2010–2011 were more likely to adopt farming adaptations, which, in turn, were associated with lower climate change risk perceptions in 2015–2016. Conversely, farmers who initially denied climate change risks tended to make fewer farming adaptations, which were associated with higher subsequent climate change risk perceptions. This suggests a complex, reciprocal relationship represented in Fig. 1 showing the Dynamic Model of Climate Action (DMCA) between climate change-related threat risk perceptions and adaptation. Individual and societal resilience could be harmed if adaptation efforts diminish over time, while climate change-related threat risks increase. Experiments establishing causal relationships between risk perceptions and adaptation would be ideal and would inform the design of conceptual models like DMCA. However, experiments beyond the hypothetical in controlled settings would be both logistically challenging and potentially unethical, as testing in a real-world context would involve assigning participants to experience repeated exposures to climate change-related threats. Instead, longitudinal studies are a powerful way to examine these relationships with larger, representative samples in real-world contexts to discern whether these processes occur throughout the populace (46).

Few studies have explored the relationship between risk perceptions and adaptation in the context of tropical cyclones (8). In a representative sample of 1,201 Americans affected by Super Storm Sandy, adapting to threats (e.g. sea-level rise) correlated with reduced intention for future adaption (47). This may reflect accurate perceptions of reduced risk: protective behavior reduces negative threat-related impacts. However, learning about the risk and ways to adapt to it also correlated with reduced judgments that impacts would occur at all. While major tropical cyclones may not make landfall in an area every year, catastrophic major storm risk is increasing globally. Thus, people must sustain adaptation like household preparedness, even without a land-falling storm. Moreover, as risks increase, individuals should ideally perform more high-effort adaptation measures such as flood proofing, which should occur prior to the next major storm. The United States (US) Gulf Coast is an ideal place to investigate the co-evolving relationship between risk perceptions and adaptation behaviors, given the stochastic nature of major land-falling storms and the familiarity of coastal residents with the risks.

The US Gulf Coast is one region expected to experience a dramatic rise in tropical cyclone damage (48). Estimates suggest that by the late 21st century (2081–2100) (49), the number of land-falling categories 4 and 5 tropical cyclones in the US will increase by more than 390%. This change will likely result in devastating impacts on life and property. Historically, tropical cyclones of this magnitude have resulted in 50% of the damage in the US, despite representing only 6% of all tropical cyclones that occur (50). Climate change may exacerbate impacts by increasing concurrent hazards such as extreme precipitation (51, 52), storms stalling over land (53), sea-level rise (10), and shifting storm locations to areas previously at low risk (54).

**Fig. 2.**
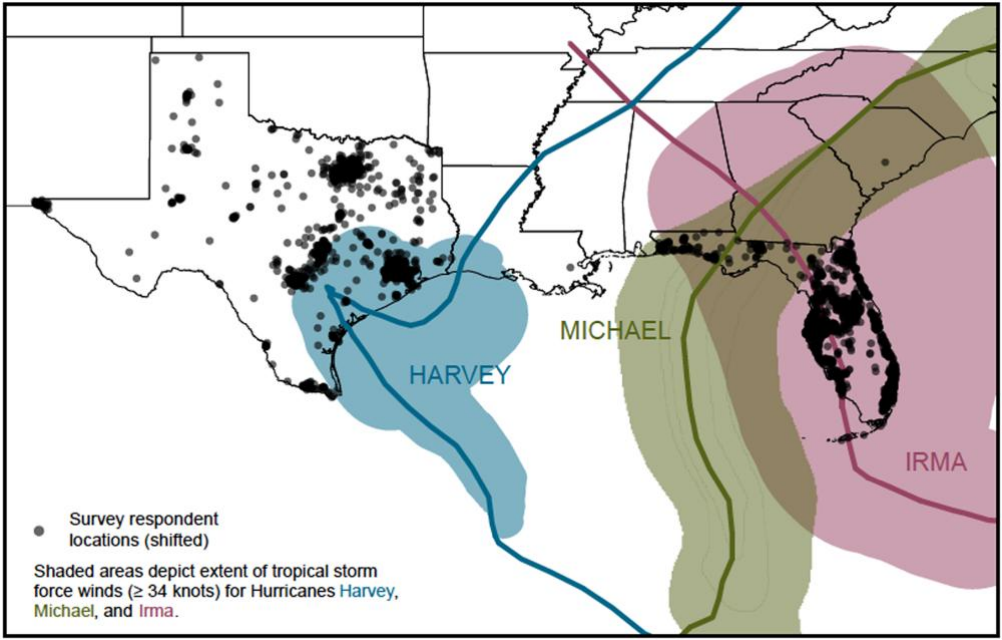
Tropical cyclone storm tracks and force winds for Hurricanes Harvey, Irma, and Michael (58–60). The solid lines represent the storm tracks for each tropical cyclone. Hurricane Harvey (2017 August 17 to 2017 September 3) resulted in over 100 deaths, catastrophic flooding, and $125 billion (2017 USD) in damage (58). Hurricane Irma (2017 August 30 to 2017 September 13) resulted in over 90 deaths and $50 billion (2017 USD) in damage (59) in the United States. Approximately 6.8 million people reported evacuating during Hurricane Irma. Hurricane Michael (2018 October 7–16) resulted in nearly 60 deaths and $25 billion (2018 USD) in damage (60). Study participants, especially those in Florida, experienced repeated exposure to major tropical cyclones during the study period. Study participants shown in this figure are those who responded to wave 1 (12). Figure created by Nina Berlin Rubin (2022).

Hence, our interdisciplinary research team surveyed a representative sample of 2,774 Texas and Florida residents 5 times on their tropical cyclone adaptation and risk perceptions as major tropical cyclones punctuated their lives between 2017 and 2022 (Fig. 2) (12, 35, 55, 56). Florida and Texas are two US Gulf Coast states that regularly experience land-falling tropical cyclones, commonly referred to as “hurricanes” in the US. From 2017 to 2022, three major land-falling tropical cyclones occurred: Hurricanes Harvey (2017), Irma (2017), and Michael (2018). Empirical literature suggests that exposure can be direct (“happened to me”), indirect (“happened to someone I know”), or through the media (12, 55, 57), and hence, a large proportion of the sample was exposed to one of these storms through one of these mechanisms over the 5-year period of the study. Residents’ risk perceptions regarding tropical cyclones and subsequent adaptation likely changed over time in the presence and absence of tropical cyclone activity.

**Fig. 3.**
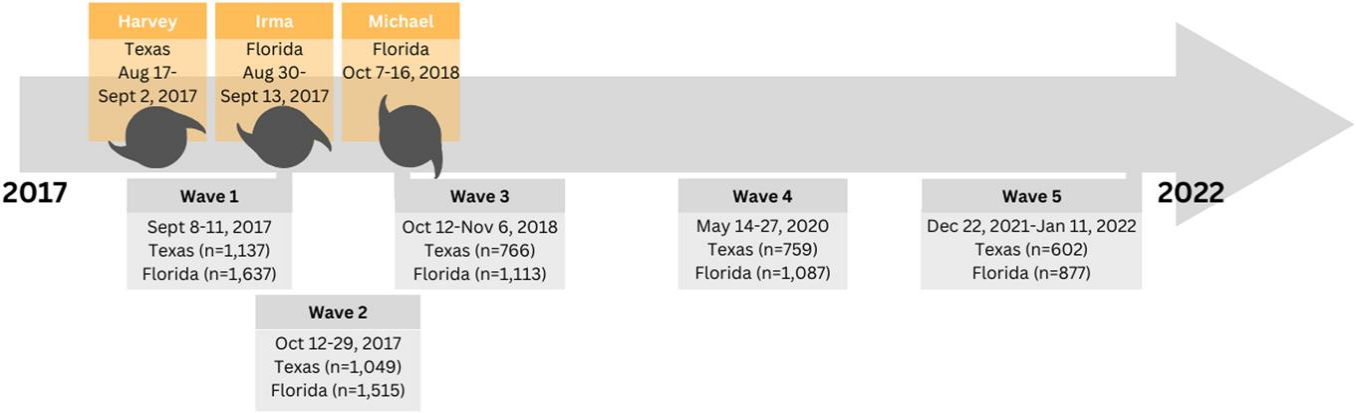
Study waves of data collection. Dates and number of participants surveyed to assess risk perceptions and adaptation behaviors between 2017 and 2022 in the presence and absence of major tropical cyclone activity (12, 35, 55, 56). While major tropical cyclones made landfall during the period between waves 4 and 5 (Hurricanes Laura (61) and Ida (62)) in the US Gulf Coast, they did not directly impact Florida and Texas. Figure created by Gabrielle Wong-Parodi (2023).

Our team recruited (12, 35, 55, 56) Ipsos KnowledgePanel panelists, sampled to be sociodemographically representative of Texas and Florida (Fig. 2). Wave 1 (12) (2017 September 8–11) occurred ∼2 weeks after Hurricane Harvey, a category 4 tropical cyclone in Texas, and in the days preceding Hurricane Irma, a category 5 tropical cyclone in Florida (Fig. 3). We then surveyed participants ∼4–6 weeks (wave 2) (12), 1 year (wave 3) (55), 2.5 years (wave 4) (56), and 4.5 years (wave 5) (56) after wave 1. We administered wave 3 in the immediate aftermath of Hurricane Michael, a category 5 tropical cyclone. We administered waves 4 and 5 in the absence of recent major tropical cyclones (see Supplementary Materials (SM), for more details).

Participants reported adaptation undertaken to prepare for the current or next tropical cyclone season at waves 3–5 using a nine-item checklist (55, 56) (e.g. putting together an emergency kit, tropical cyclone-proofing home). Participants shared personal tropical cyclone risk perceptions by expressing expected chances (0–100%) of home damage and injury on a four-item measure (63) at waves 1–3 (12) and 5 (56). Participants reported their future tropical cyclone risk perceptions at waves 3 (55) and 5 (56), using a two-item measure assessing the expected intensity and frequency (less, about the same, or more) of future storms. Participants reported lifetime negative experiences related to tropical cyclones they may have been exposed to at wave 1 and updated responses at waves 2 and 3 (Fig. 1) (12, 55), using a six-item list (35) that included property damage, injury to self, or a loved one's death or injury (see Materials and Methods and SM, for more details).

We hypothesized (H1) that as tropical cyclone adaptation increased over time, personal tropical cyclone risk perceptions in terms of home damage and injury would decrease. Since negative experiences positively correlate with risk perceptions (35, 36), we further hypothesized (H2) the association between tropical cyclone adaptation and personal tropical cyclone risk perceptions would be moderated by negative tropical cyclone experiences; more negative experiences would be associated with greater personal risk perceptions, whereas fewer negative experiences would be associated with lower personal risk perceptions.

Since adapting may depress perceptions of the chances of tropical cyclone impacts (47), we had two additional exploratory research questions. RQ1: Do future tropical cyclone risk perceptions (frequency and severity) decrease as tropical cyclone adaptation increases over time? RQ2: Is the association between tropical cyclone adaptation and future tropical cyclone risk perceptions moderated by negative tropical cyclone experiences?

Our study contributes four major insights: (i) personal tropical cyclone risk perceptions declined over time—especially in the absence of tropical cyclone activity, yet different patterns of perceptions emerged; (ii) most reported that tropical cyclones in the future will have about the same or less frequency and intensity, with views being stable over time; (iii) personal tropical cyclone risk perceptions positively correlated with tropical cyclone adaptation, yet performing more adaptation was associated with decreasing personal risk perceptions; and (iv) more negative tropical cyclone experience positively correlated with personal but not future tropical cyclone risk perceptions.

## Results

### Trends and patterns of personal tropical cyclone risk perceptions

**Fig. 4.**
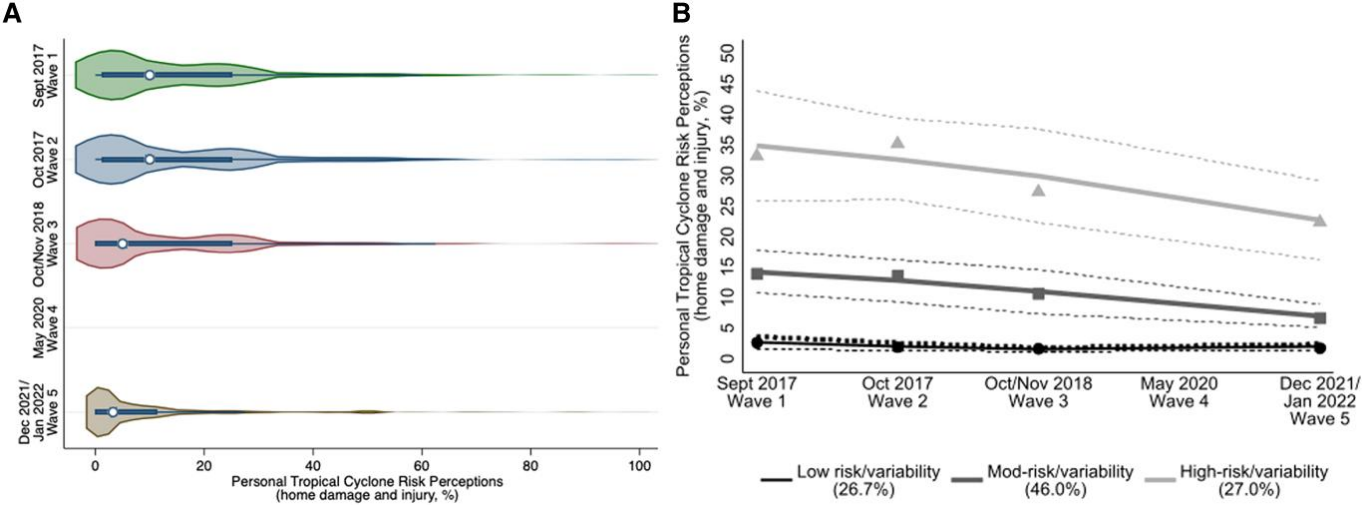
Personal tropical cyclone risk perceptions by wave (panel A) and average personal tropical cyclone risk perceptions by group and by wave (panel B). Panel A shows the quartiles and density (frequency) of participant responses regarding personal tropical cyclone risk perceptions. Personal tropical cyclone risk perceptions decreased over time, especially in the absence of storm activity in Florida and/or Texas. Note that participants were not asked about personal tropical cyclone risk perceptions at wave 4. Panel B shows the data-driven latent group-based trajectories of personal tropical cyclone risk perceptions over time (censored normal model; solid lines = estimated trajectories; dot symbols = observed group means at each wave; % = estimated group percentages; dotted lines = 95% CI). Note the *y*-axes are rescaled to 50%; see SM for figures at full scale. Personal hurricane risk perceptions ranged from 0 to 100%. Data used for A and B are only for those participants who did not drop out at any time across waves.

Personal tropical cyclone risk perceptions declined, on average, 2.50% between waves 3 and 5 (SE = 0.43, *P* < 0.001, Fig. 4A, Table S11). Closer inspection revealed the lowest perceptions of risk (9.38% chance of home damage and injury) during periods of no tropical cyclone activity in Florida and Texas, despite there being recent major land-falling tropical cyclones in neighboring states. The highest risk perceptions (12.58–16.37%) occurred during periods of tropical cyclone activity in Florida and/or Texas, respectively (Fig. S7). This pattern of decay in risk perceptions over time has been observed in other threat domains, including financial crises (64) and extreme weather events like floods (65). Explanations include the availability heuristic (66)—how easily people conceptualize examples when judging the likelihood of a future event. Extending these findings to the domain of tropical cyclones, our longitudinal design with data collected in the presence and absence of storms allows us to explore *variability* in the trajectories of risk perceptions. Understanding this variability offers insight into more effective adaptation interventions for different risk perception profiles, given observed positive associations between risk perceptions and adaptation (4) (see Tables S11 and S12 and Fig. S6, for more details).

We used data-driven, group-based trajectory modeling to identify three distinct patterns of personal tropical cyclone risk perception trajectories: low risk (*n* = 497, 26.70% of participants), moderate risk (*n* = 707, 46.00% of participants), and high risk (*n* = 272, 27.00% of participants). Of the sociodemographic covariates included in the modeling, those without a college degree and those living in Florida (compared with Texas) were more likely to be in the high-risk than the low-risk group.

Making up about one-quarter of the sample, the low-risk group's personal tropical cyclone risk perception trajectory was around 0% irrespective of storm activity (Fig. 4B). This low-risk group may have previously adapted and thus expressed lower risk perceptions; however, we observed a positive association between risk perceptions and adaptation on average (*b* = 1.68, SE = 0.23, *P* < 0.001), indicating this explanation is unlikely. Low risk perceptions may also be explained by the “near miss” effect (67–69)—a potentially disastrous tropical cyclone did not occur, resulting in an inflated perception of personal resiliency and associated low risk perceptions. This trajectory may also be explained by the “optimism bias” (70)—the tendency to overestimate the likelihood of good events and underestimate the likelihood of bad events. Another explanation for this trajectory could be a memory effect, where people respond consistently to repeated questions because they recall their previous responses. This is unlikely as the length of time between surveys ranged from 4 to 6 weeks to multiple years, far beyond the relatively short time (tens of minutes) assessed for accurate recall in the literature (71, 72).

Representing nearly half of the sample, the moderate-risk group's personal tropical cyclone risk perceptions were higher and more variable than the low-risk group, about 15% initially higher during tropical cyclone activity and declining in its absence (Fig. 4B). Comprising about one-quarter of the sample, the high-risk group's tropical cyclone risk perceptions were like the moderate-risk group but more extreme, about 32% initially higher than the low-risk group (Fig. 4B). The observed increase in personal tropical cyclone risk perceptions for this group in the weeks following Hurricanes Harvey and Irma may be due to living and dealing with the tropical cyclone aftermath (73)—e.g. property and income loss, illness. These perceptions precipitously decreased after another major tropical cyclone (Hurricane Michael), which impacted far fewer people, ultimately leveling off in the absence of other storms (years 2020–2022). Similar to previous studies (74, 75), we found those without a college degree were more likely to be in the high-risk group than the low-risk group. Those with less education may have less access to resources and existing power structures (74), and they may be more vulnerable to disasters, including major tropical cyclones (see Tables S13 and S14, and Figs. S10 and S11, for more details).

### Future tropical cyclone risk perceptions

**Fig. 5.**
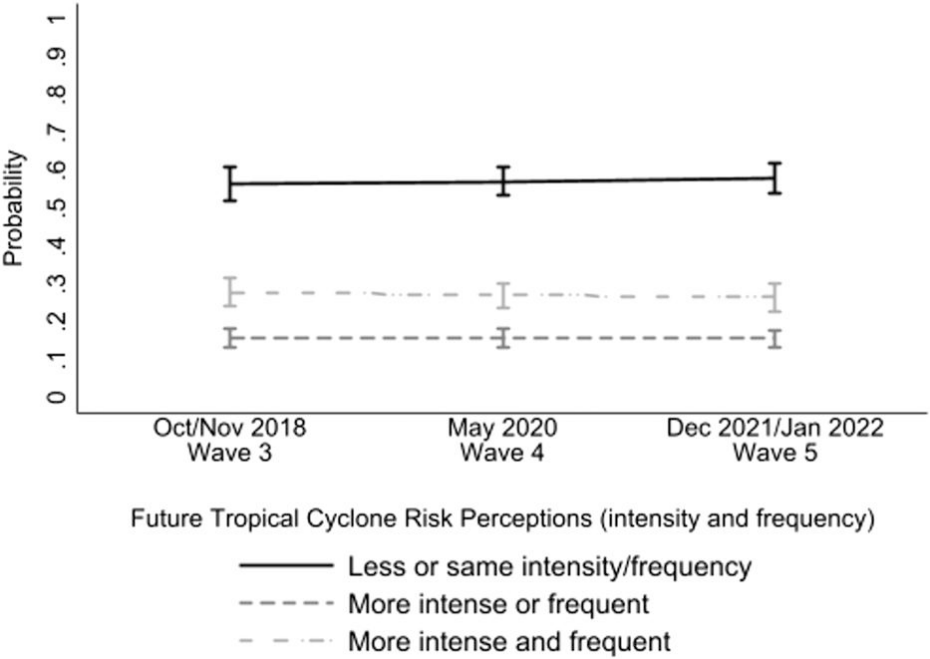
Future tropical cyclone risk perceptions over time. Figure shows the main effect of time on future tropical cyclone risk perceptions controlling for self-reported tropical cyclone adaptation and covariates. Bars represent 95% CI. Model includes wave 5 poststratification survey weights.

Most participants (56.51% at wave 3) reported that tropical cyclones in the future will have about the same or less intensity and frequency as they have today, and these perceptions remained stable over time (58.54% at wave 5, *P* = 0.345; Fig. 5). Evidence suggests that recent experience with the physical parameters of tropical cyclones, including high wind speeds, is strongly and positively associated with perceptions about changes in tropical cyclone intensity (76). We first asked participants about their future tropical cyclone risk perceptions after Hurricane Michael, which affected some respondents, and then several years later, in the absence of a major storm. The availability heuristic may explain this prevalent view that intensity and frequency of future storms will be the same or be less in the future. Views about climate change have also been correlated with views about the likelihood of future weather extremes like wildfires (77) and tropical cyclones (35). Given the politicization regarding climate change in the United States, asking participants about the future intensity and magnitude may have triggered a polarized response: those who view climate change as happening may see future tropical cyclone risks increasing, whereas those who do not see it as happening may see risks staying the same or decreasing. This finding may also be due to unclear communication regarding climate change and tropical cyclone risk: intensity, but not frequency, of major storms is predicted to increase due to climate change (49) (see Table S17, for more details).

### Adaptation and personal tropical cyclone risk perceptions over time

**Fig. 6.**
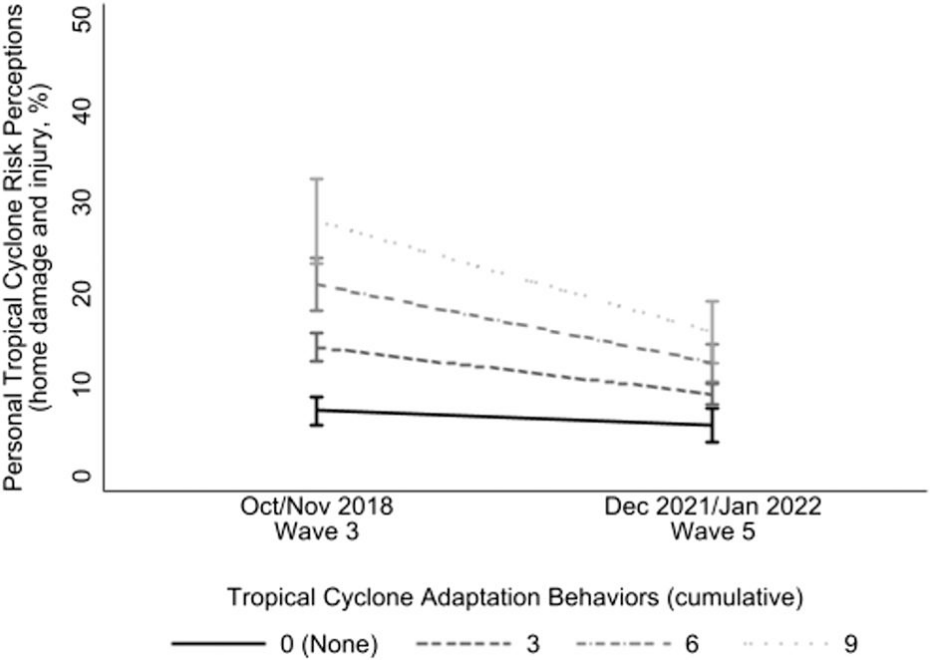
Association between personal tropical cyclone risk perceptions and tropical cyclone adaptation (cumulative) over time. Figure shows that, controlling for demographic covariates, as tropical cyclone adaptation increased, personal tropical cyclone risk perceptions decreased over time (bars = 95% CI). Note the *y*-axes are rescaled to 50%; see SM for figures at full scale. Personal hurricane risk perceptions ranged from 0 to 100%. Model included wave 5 poststratification survey weights.

As hypothesized, across waves, for each additional tropical cyclone adaptation between waves 3 and 5, participants reported a 1.68% increase in personal tropical cyclone risk perceptions (SE = 0.23, *P* < 0.001; Fig. S6). Yet importantly, over time, the association between adaptation behaviors and personal risk perceptions became weaker in the absence of tropical cyclone activity (*b* = −0.59, SE = 0.16, *P* < 0.001; Fig. 6). Those reporting the performance of nine tropical cyclone adaptations had more dramatic decreases in personal risk perceptions (a 6.09% decrease, SE = 1.21, *P* < 0.001) than those who reported no adaptation (a 0.79% decrease, SE = 0.49, *P* = 0.106). Adaptation was measured cumulatively, which might explain this observed relationship. Yet these findings are consistent with the rare studies examining this relationship cross-sectionally (47) and longitudinally (6). It is also consistent with a growing body of evidence in the field of public health finding a positive relationship between health protective behaviors and risk perceptions (78); particularly, if the protective behavior is viewed as being effective (79). This longitudinal association was insensitive to negative tropical cyclone experiences. Moreover, performing tropical cyclone adaptations was unrelated to future tropical cyclone risk perceptions.

This study's representative, longitudinal design sheds light on the dynamic co-evolving relationship between risk perceptions and adaptation in the context of tropical cyclones, a climate change-related threat. On average, personal tropical cyclone risk perceptions were positively associated with adaptation, as previously observed (4). Yet when people continued to adopt such behaviors over time, their perceptions of personal risk decreased. This change in risk perceptions is sensible: when risk-reducing behaviors are adopted, personal risk should decrease. Yet the chances of more intense tropical cyclones are increasing (49), with likely devastating impacts on life and property (80). This requires sustained action. Moreover, the most common tropical cyclone adaptation reported was putting together an emergency supply kit or learning about the risks and ways to prepare (Fig. S2). While helpful, these are arguably less protective than making ones’ home more tropical cyclone proof or purchasing flood insurance (24, 25), which were reported less frequently (see Table S12 and Figs. S8 and S9, for more details).

### Negative experience and tropical cyclone risk perceptions

**Fig. 7.**
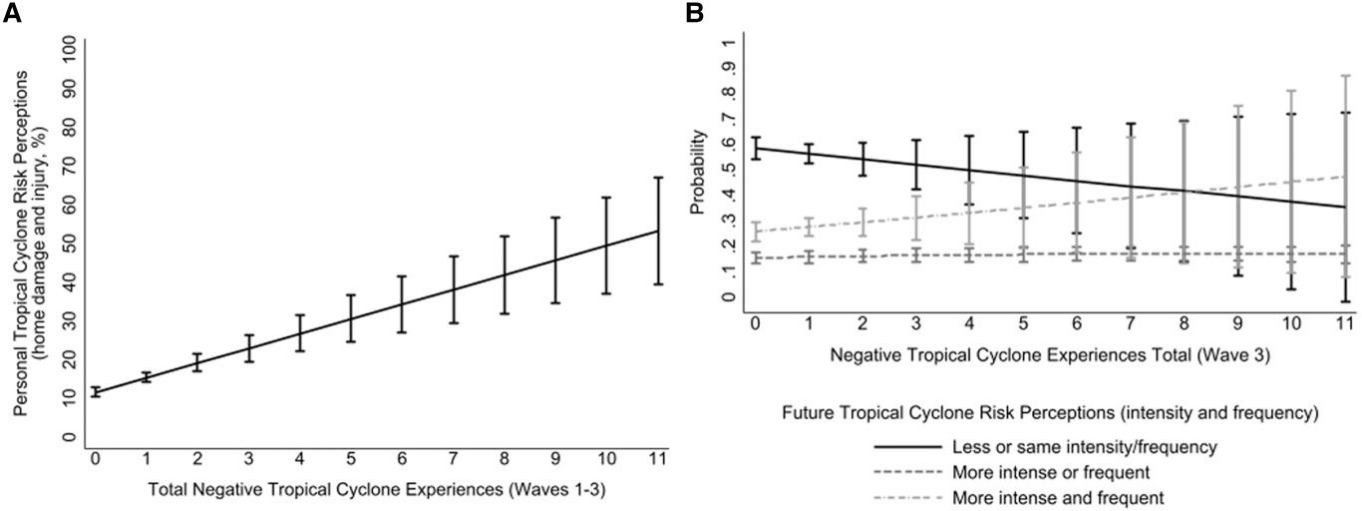
Personal (panel A) and future (panel B) tropical cyclone risk perceptions associated with negative tropical cyclone experiences. Panel A shows the main effect of self-reported negative tropical cyclone experiences (waves 1–3) and personal tropical cyclone risk perceptions (home damage and injury %), controlling for time, self-reported tropical cyclone adaptation (cumulative), and covariates. Panel B shows the main effect of self-reported negative tropical cyclone experiences (waves 1–3) and future tropical cyclone risk perceptions controlling for time and covariates. Models included wave 5 poststratification survey weights. Bars represent 95% CI.

More negative tropical cyclone experiences were associated with greater personal *and* future tropical cyclone risk perceptions. For each additional negative tropical cyclone experience (e.g. home damage, personal injury), participants reported a 3.78% increase in personal tropical cyclone risk perceptions (SE = 0.66, *P* < 0.001, Fig. 7A) and a 0.09 unit increase in future tropical cyclone risk perceptions (SE = 0.08, *P* = 0.280; Fig. 7B). These findings add support to the balance of the literature, suggesting that experiences with extreme weather, especially those that are negative (35, 36), are positively associated with greater risk perceptions in terms of personal risk (see Tables S13–S15, S19, and S20, for more details).

## Discussion

Taken together, evidence from this representative, longitudinal study of Florida and Texas residents suggests tropical cyclone risks are generally considered in the context of recent or anticipated near-term events. This is seen by a sensible decline in personal risk perceptions (“Taking action to prepare my household for damage and injury will reduce the risk of damage and injury”), which are unrelated to future risk perceptions. On average, participants did not perceive the future risk of tropical cyclones as increasing, with some reporting decreasing risk over time. Moreover, we observed few participants electing to make their home more tropical cyclone proof or having flood insurance, which would offer more future protection than putting together a first aid kit (4, 23–25). Our findings suggest that adaptation for salient climate change-related threats may counterintuitively lower personal risk perceptions and leave people less prepared for future threats.

Strengths of our study include its longitudinal design with a large representative sample of individuals from Florida and Texas who were repeatedly surveyed over a period of 5 years. We also note some limitations. First, not everyone that we invited to take the initial survey accepted our invitation; the response rate was 46.70%. Therefore, there may have been differences in tropical cyclone risk perceptions among those who elected to take the survey vs. those who did not. However, the response rate we achieved is comparable with other similar studies (12, 55). Moreover, our primary interest was in understanding individual change over time, and we achieved very high rates of retention wave over wave: 86.62% at wave 2, 70.19% at wave 3, 73.63% at wave 4, and 83.75% at wave 5. Second, some participants may not have been in their community during tropical cyclone events or seasons; hence, potentially affecting their expressed risk perceptions. Nevertheless, we included only those participants who maintained continuity of community of residence throughout the study period, as verified by Ipsos. Future studies may want to assess the actual location of participants during periods of climate change-related threat exposure. Third, as many of the adaptations were durable goods and reusable, we assumed that people who indicated once they had these items (i.e. a rowboat) retained them for the duration of the study period, even if they did not check the item at a subsequent wave. Indeed, evidence suggests that previous adaptive actions predict subsequent actions (81). Nevertheless, it could be the case that participants did not continue with certain adaptations (i.e. flood insurance or having a rowboat) over time. Future studies may want to more specifically assess behaviors that were both newly initiated and sustained over time. Fourth, while our longitudinal design allowed for understanding co-evolving relationships over time, we cannot investigate causality. Future experimental or quasi-experimental (e.g. discontinuity design) studies may be employed to better understand causal relationships.

Evidence from this representative, longitudinal study of Florida and Texas residents exposed to major tropical cyclones suggests that despite the benefits of adapting, such behaviors may paradoxically reduce the perceived threat of climate change-related threat impacts, potentially suppressing future adaptation as threats escalate. Policies and programs that support investment in long-term, proactive household- and community-level adaptations are warranted, especially among those who may be less well-resourced or supported.

## Materials and methods

### Recruitment

Participants were recruited from Ipsos KnowledgePanel (formerly GfK). Ipsos uses address-based sampling to randomly recruit panelists using probability-based sampling methods: the panel is designed to be representative of the United States. Ipsos uses the Delivery Sequence File of the United States Postal Service, which includes all delivery point addresses, when sampling. This approach improves population coverage relative to traditional random-digit dialing methods, enabling recruitment of harder-to-reach individuals, such as younger people. Ipsos provides those households who do not have internet access with a web-enabled device and free Internet services. Once household members are recruited for the panel and assigned to a study, they are notified electronically of the opportunity. Panelists can then take the survey either through their email link or by visiting their online member page.

For this study, participants were invited to take surveys on five separate occasions (12, 55, 56). Wave 1 was fielded 2017 September 8–11, weeks after Hurricane Harvey and days before Hurricane Irma (12). Wave 2 was fielded 2017 October 12–29, 4–6 weeks after wave 1 (12). Wave 3 was fielded 2018 October 22 to November 6, about 1 year after wave 1 and weeks after Hurricane Michael (55). Wave 4 was fielded 2020 May 14–27, about 2.5 years after wave 1 (56). Finally, wave 5 was fielded 2021 December 20 to 2022 January 11, about 4.5 years after wave 1 (56). Each survey took between 10 and 12 min to complete, and participants were compensated $20 for completing each survey. After wave 1, response rates were high (73.6–86.6%) over time (see Table S3, for more details).

### Survey weights

While Ipsos takes great care to recruit a representative sample of US adults, weights are created to ensure that all samples are representative. Geodemographic benchmarks identified from the latest March Current Population Survey (82) are as follows: sex, age, race/ethnicity, household income, metro/non-metro, and education. For this study, we used study-specific poststratification weights to ensure that respondents from Texas and Florida from the KnowledgePanel are representative of the adult populations of these states.

### Institutional review board

The study received institutional review board approval from the authors’ institutions (Stanford University, IRB-52533, and the University of California, Irvine, IRB-2016-2827). All participants provided informed consent when they enrolled in the panel, and the researchers provided a study information sheet before each survey.

### Survey measures

#### Adaptation

In waves 3–5 surveys (55, 56), participants indicated the adaptations they adopted to prepare for the present, past, or future hurricane season: (i) “Learn more about the risks from hurricanes and how to prepare for them,” (ii) “Put together an emergency kit (e.g. food, medical supplies, flashlight),” (iii) “Develop and practice an emergency plan,” (iv) “Identify shelter locations in the event of an evacuation,” (v) “Copy important documents (e.g. birth certificates, driver’s licenses),” (vi) “Get a row boat or inflatable raft,” (vii) “Make my home more hurricane proof (e.g. install hurricane shutters, sand bags),” (viii) “Have flood insurance,” and (ix) “Other (please specify).” At wave 3, a count score was calculated by summing across the adaptations selected by participants. At wave 4, a cumulative count score was calculated by summing across the adaptations selected by participants and adding those behaviors selected at wave 3 but not at wave 4. At wave 5, a cumulative count score was calculated by summing across the adaptation behaviors selected by participants and adding those behaviors selected at wave 3 or 4 but not at wave 5. We elected to create count scores in this way as we assumed many of the items listed above were durable and reusable. For example, there is reason to believe that participants who purchase a boat for hurricane safety purposes at wave 4 will still be able to use it at wave 5. For items that are not durable and reusable, while this is not the case for all behaviors, evidence suggests that previous adaptations positively correlate with subsequent actions (81) (see Tables S3 and S4 and Fig. S1, for more details).

#### Personal risk perceptions

In waves 1–3 (12, 55) and 5 (56) surveys, participants completed a four-item measure of personal risk perceptions regarding the chances of harm from a hurricane or its aftermath. Two were about home damage: (i) “Your home will be severely damaged or destroyed because of a hurricane or its aftermath” and (ii) “You will never be able to return to your current home as a result of a hurricane or its aftermath” (0–100%). Two were about injury: (iii) “You will be seriously injured by a hurricane or its aftermath” and (iv) “Someone close to you will be seriously injured by a hurricane or its aftermath” (0–100%). Responses were averaged at each wave (see Table S5 and Fig. S3, for more details).

#### Future risk perceptions

In waves 3 (55) and 5 (56) surveys, participants completed a two-item measure of future risk perceptions regarding hurricane intensity and frequency in future hurricane seasons: (i) “Do you think hurricanes during future hurricane seasons will be” (−1 = less intense than the ones in prior hurricane seasons, 0 = about the same intensity, 1 = more intense than the ones in prior hurricane seasons) and (ii) “Do you think hurricanes during future hurricane seasons will” (−1 = happen less frequently than the ones in prior hurricane seasons, 0 = happen with about the same frequency, 1 = happen more frequently than the ones in prior hurricane seasons). These items were combined and recoded as follows: 0 = less or about the same intensity and frequency as prior hurricane seasons, 1 = either more intensity or frequency as prior hurricane seasons, and 2 = more intensity and frequency as prior hurricane seasons (see Table S6 and Fig. S4, for more details).

#### Negative experiences

In waves 1–3 (12, 55) surveys, participants reported on a six-item measure if they had negative hurricane-related experiences because of Hurricanes Harvey, Irma, and/or Michael: (i) “I lost property in the hurricane or its aftermath,” (ii) “My home was totally destroyed in the hurricane or its aftermath,” (iii) “I was injured in the hurricane or its aftermath,” (iv) “I lost a pet in the hurricane or its aftermath,” (v) “I knew someone who was injured in the hurricane or its aftermath,” and (vi) “I knew someone who was killed in the hurricane or its aftermath.” One cumulative count score was calculated by summing all reports of negative experience across the three waves (see Table S7 and Fig. S5, for more details).

### Data analysis

Stata version 17.0 was used for all analyses (83). Mixed-effects modeling was conducted to examine the interaction between time and tropical cyclone adaptation behaviors on personal tropical cyclone risk perceptions and future tropical cyclone risk perceptions, as well as the interaction between time and negative tropical cyclone experiences, and adaptation behaviors and negative tropical cyclone experiences, each on personal risk perceptions.

In terms of fixed effects, each model included age, gender, race/ethnicity, household income, education, and state (TX = 0, FL = 1) as time-varying covariates. For random effects, each model began with only by-participant random intercepts. Model estimates were stored, and Akaike Information Criterion (AIC) and Bayesian Information Criterion (BIC) were calculated. Then, random slopes for the main predictor were added to the model. A likelihood ratio test was conducted to determine whether the addition of the random slopes significantly improved the model. All mixed-effects models used wave 5 poststratification survey weights, included by-participant random intercepts, used unstructured covariance matrix, allowed correlation among random effects, and were estimated using a maximum-likelihood approach. Main effects were estimated for each model by removing the time by the main predictor interaction term.

A linear mixed-effects model was constructed predicting personal tropical cyclone risk perceptions (home damage and injury %) from the interaction between time and cumulative self-reported tropical cyclone adaptation behaviors count controlling for covariates with by-participant adaptation behaviors random slopes. Results from the LRT showed that adding the random slopes of adaptation behaviors (AIC = 21,521.02, BIC = 21,610.57) significantly improved the model over the intercepts-only model (AIC = 21,605.43, BIC = 21,684.45), *χ*^2^ (2, *n* = 1,433) = 88.42, *P* < 0.001. From there, we tested including by-participant time random slopes; however, the model failed to estimate standard errors for the random effect. The model failed to converge when including the interaction random slopes of time × adaptation behaviors. Thus, the model with by-participant random intercepts and adaptation behaviors random slopes was retained.

To further explore personal tropical cyclone risk perceptions over time, group-based multitrajectory modeling was conducted. Specifically, a finite (discrete) mixture model for clustering longitudinal data was constructed using the *traj* package (84, 85) in Stata. This method applies a discrete mixture model of multiple latent classes to identify relatively homogeneous clusters of trajectories over time using maximum likelihood estimation (86). The optimal model (number of classes) was determined based on a two-stage approach (87) comparing BIC (lower absolute values suggest better model fit), entropy (closer to 1.0 suggests clearer delineation among classes), and visual inspection of trajectory interpretability. All models were censored normal, used wave 5 poststratification survey weights, controlled for wave 5 age, gender (male = 0, female = 1), race/ethnicity, education, household income, state (Texas = 0, Florida = 1), and wave 3 cumulative self-reported negative tropical cyclone experiences count, and estimated bootstrapped 95% CIs with 10,000 repetitions. The *traj* command uses a robust (sandwich) estimator of the variance-covariance matrix when survey weights are used. This analysis was not preregistered.

For the first stage, the number of latent groups was tested using the cubic form for all trajectory groups (models using quartic polynomials failed to estimate the covariance matrix due to nonsymmetry or high singularity). Based on our data and research questions, we tested models with 2, 3, and 4 latent classes, and model fit is summarized in Table S12. The four-class solutions produced two-class trajectories that largely overlapped, making interpretability difficult. BIC and entropy estimates suggested that a two-class solution fit best, although a third interpretable class clearly emerged. Thus, we continued to the second stage, examining models with two and three latent classes.

The second stage consisted of determining the order of the polynomial functions to specify the shape of each trajectory. Each order of polynomials (linear, quadratic, and cubic) was tested for models with two and then with three latent classes. Model fit is summarized in Table S13. A two-class solution with cubic and quadratic trajectories emerged with the best fit. For models with three classes, two had similar BIC and entropy values. The model with three cubic polynomial functions was retained. For the final model, we estimated for each participant the posterior predicted probability of belonging to each latent class. Participants were assigned to the latent class in which their predicted probability was largest. The adequacy of the model was next assessed by estimating the average posterior probability (APP) of assignment for each group. Based on the recommended 0.70 threshold, our model showed acceptable discrimination in classifying individuals into distinct trajectory classes: APP class 1 = 0.8711, class 2 = 0.8799, and class 3 = 0.9223. Odds of correct classification (OCC) using the weighted posterior proportions were also estimated, and each was above the 5.0 recommended threshold (5) for demonstrating assignment accuracy: OCC class 1 = 14.67, class 2 = 8.01, and class 3 = 45.45.

A linear mixed-effects model was constructed predicting personal tropical cyclone risk perceptions (home damage and injury %) from the interaction between time and self-reported negative tropical cyclone experiences total count (wave 3) controlling for covariates and with negative tropical cyclone experiences random slopes. Results from the LRT showed that adding the negative tropical cyclone experiences random slopes significantly improved the model (AIC = 45,453.64, BIC = 45,543.69) over the intercepts-only model (AIC = 45,479.18, BIC = 45,558.64), *χ*^2^ (2, *n* = 1,476) = 29.54, *P* < 0.001. Next, a by-participant random slope for time was added to the existing model; however, this did not improve model fit, and the model failed to produce random effects variance. Thus, the model with by-participant random intercepts and negative tropical cyclone experiences random slopes as random effects was retained. The model was then repeated by replacing the time interaction term with a negative tropical cyclone experience *×* tropical cyclone adaptation behaviors interaction.

An ordered probit mixed-effects model was constructed predicting future tropical cyclone risk perceptions (intensity and frequency) from the interaction between time (continuous) and cumulative self-reported tropical cyclone adaptation behaviors controlling for covariates with by-participant adaptation behaviors random slopes. Results from the LRT showed that adding the adaptation behaviors random slopes (AIC = 4,776.43, BIC = 4,866.02) significantly improved the model over the intercepts-only model (AIC = 4,788.43, BIC = 4,867.48), *χ*^2^ (2, *n* = 1,437) = 16.00, *P* < 0.001. The model failed to converge when including an additional by-participant time random slope; thus, the model with by-participant random intercepts and adaptation behaviors random slopes as random effects was retained.

Lastly, an ordered probit mixed-effects model was constructed predicting future tropical cyclone risk perceptions (intensity and frequency) from the interaction between time (continuous) and cumulative self-reported negative tropical cyclone experiences total count (wave 3), controlling for covariates with by-participant negative experiences random slopes. Results from the LRT showed that adding negative tropical cyclone experiences random slopes (AIC = 4,861.93, BIC = 4,951.98) significantly improved the model over the intercept-only model (AIC = 4,869.57, BIC = 4,949.04), *χ*^2^ (2, *n* = 1,476) = 11.64, *P* = 0.003. Thus, the model with by-participant random intercepts and negative tropical cyclone experiences random slopes was retained.

## Supplementary Material

pgae099_Supplementary_Data

## Data Availability

Anonymized data for waves 4 and 5 will be placed on the Inter-university Consortium for Political and Social Research repository upon publication. Anonymized data can be made available upon request from researchers who have undergone ethics review for waves 1–3.

## Preprints

The authors preregistered the analyses at Open Science Forum (osf.io/d2xtw) unless otherwise indicated.
