# Supplementary material for: A longitudinal investigation of risk perceptions and adaptation behavior in the US Gulf Coast
Source: PNAS Nexus. 2024 Apr 9;3(4):pgae099. doi: 10.1093/pnasnexus/pgae099 (PMC11003376; doi:10.1093/pnasnexus/pgae099)
Supplement: pgae099_Supplementary_Data [file pgae099_supplementary_data.pdf]

Supplementary Information Appendix for

A probability-based longitudinal investigation of risk perceptions and adaptation behavior in the US Gulf Coast

Gabrielle Wong-Parodi *et al.*

*Corresponding author. Email: gwongpar@stanford.edu

This PDF file includes:

Figs. S1 to S11
Tables S1 to S20

Figure S1.

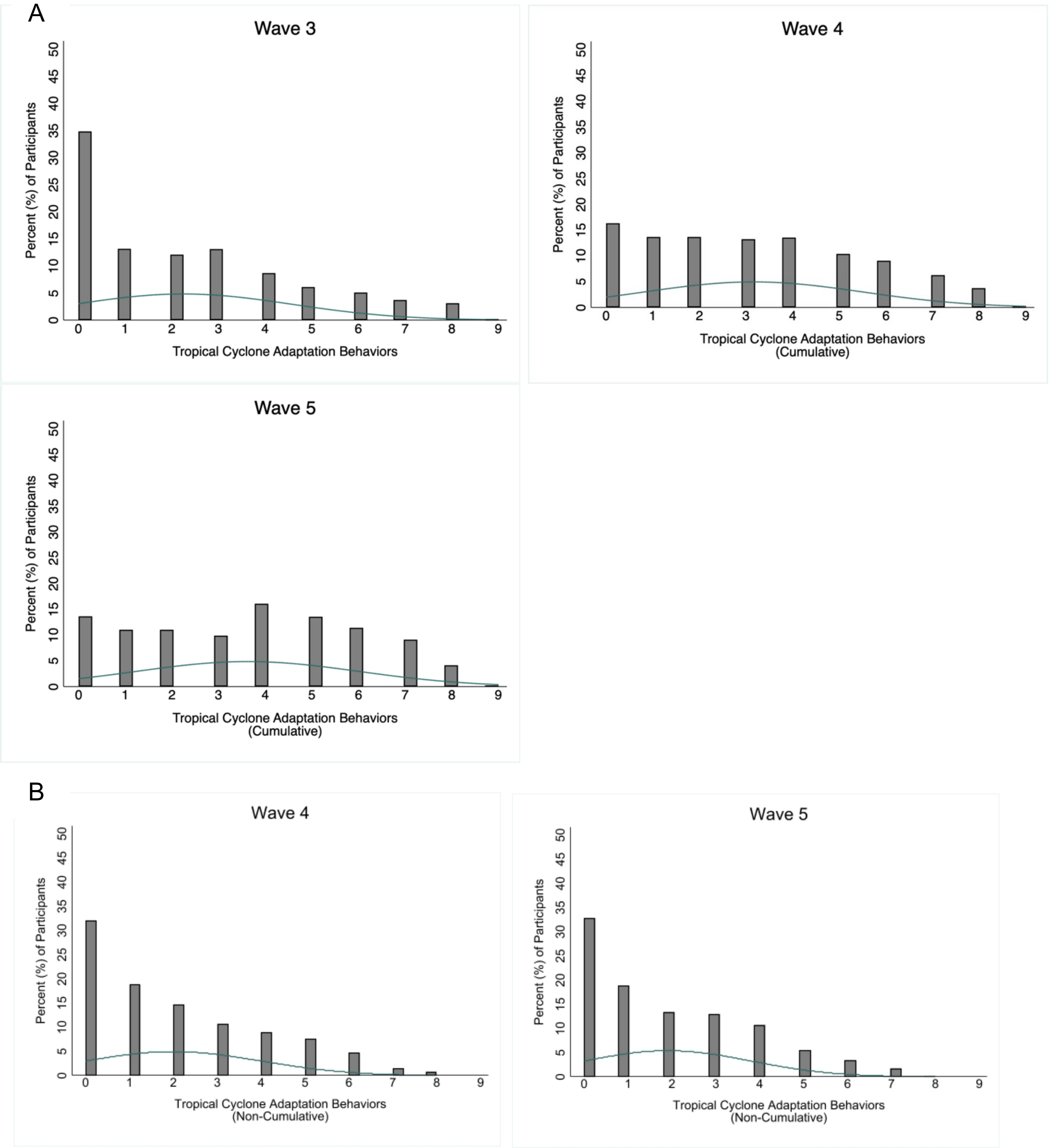

(A) Histograms of cumulative self-reported personal tropical cyclone (hurricane) adaptations taken over time. Wave 4 shows adaptations taken at both Wave 3 and new reported behaviors. Wave 5 shows adaptations taken at Waves 3 and 4 and new reported behaviors. (B) Histograms of non-cumulative self-reported personal tropical cyclone (hurricane) adaptations taken over time. Note the y-axis in these figures is rescaled from a total of 100%. Line = normal density curve. Each histogram is weighted to its own wave survey poststratification weights (e.g., Wave 4 includes all participants who completed up to Wave 4).

Figure S2.

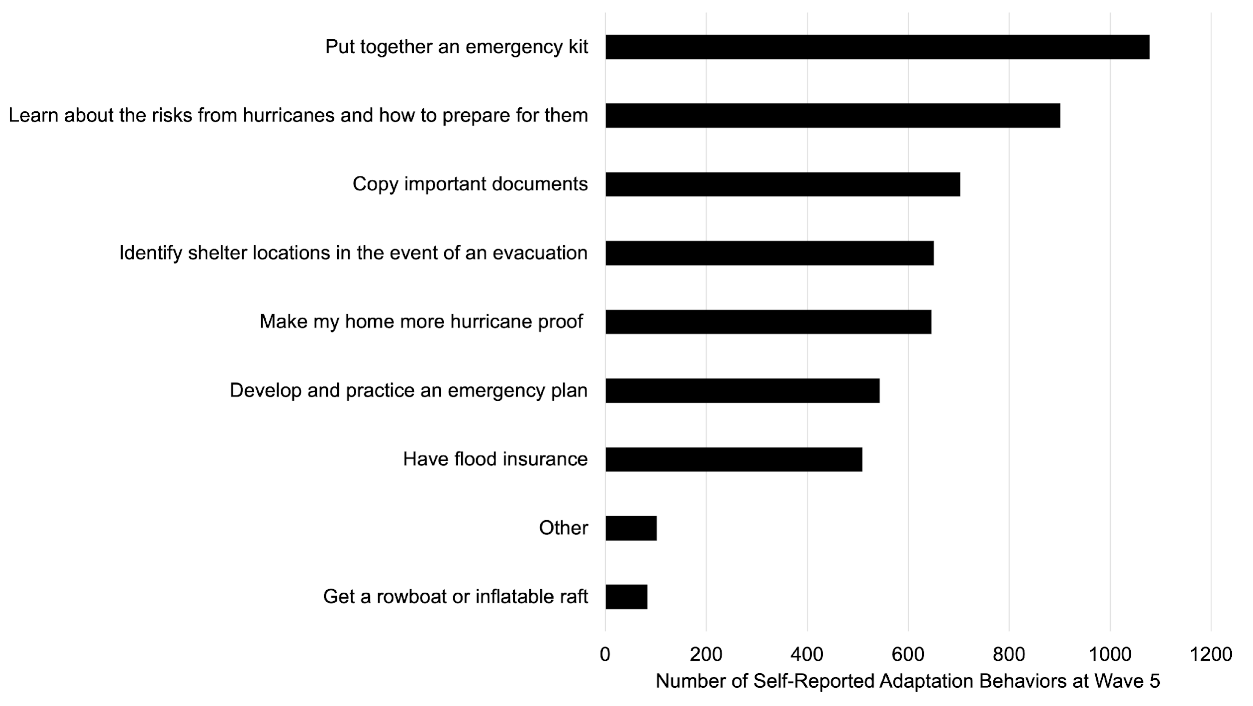

Number and type of tropical cyclone (hurricane) adaptations self-reported at Wave 5. Wave 5 poststratification survey weights were applied.

Figure S3.

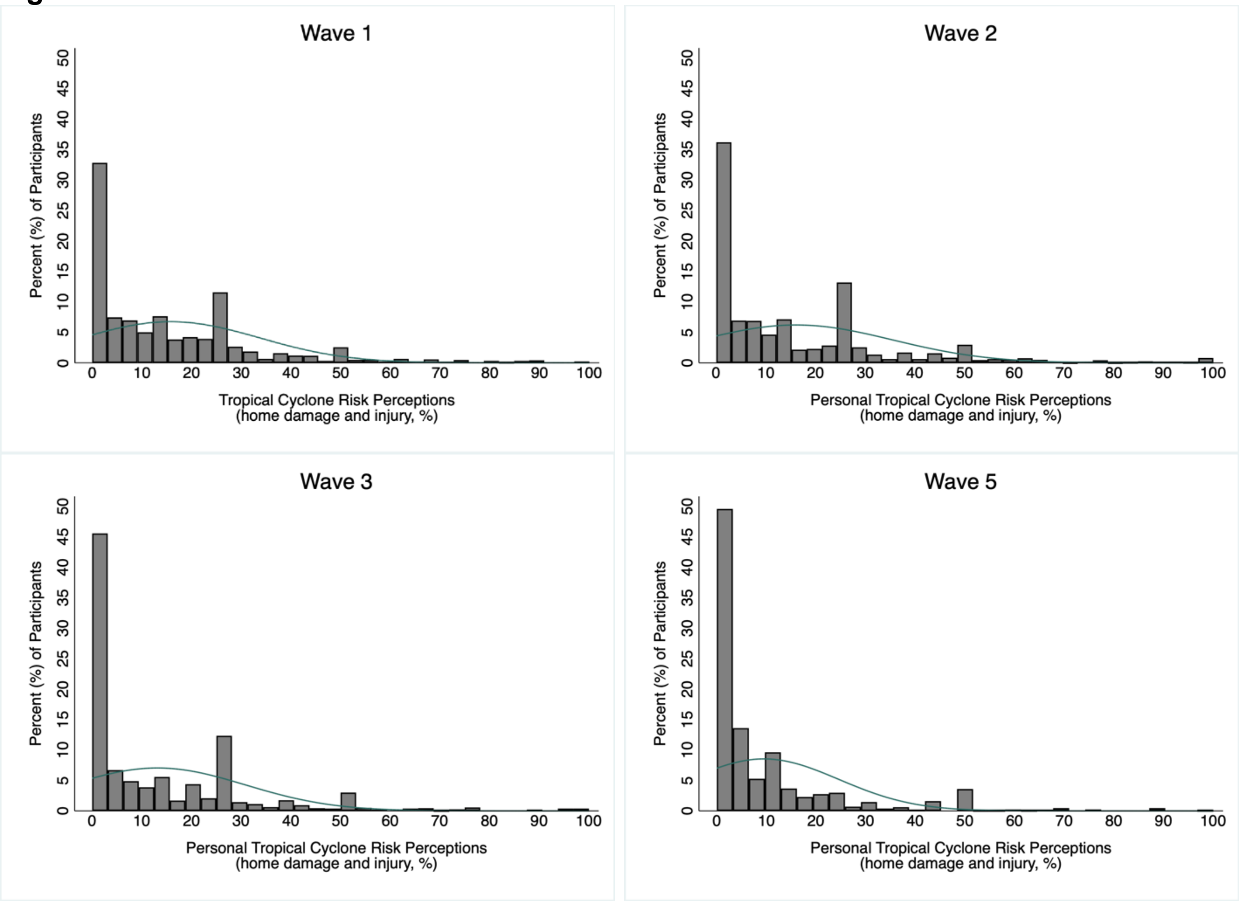

Histograms of personal tropical cyclone (hurricane) risk perceptions (% chance of home damage and injury to self and close others) at Waves 1-3 and 5. Note: the y-axes in these figures are rescaled from a total of 100%. Each histogram is weighted to its own wave survey poststratification weights (e.g., Wave 4 includes all participants who completed up to Wave 4, Wave 5 illustrates data from participants who completed all waves). Line = normal density curve.

Figure S4.

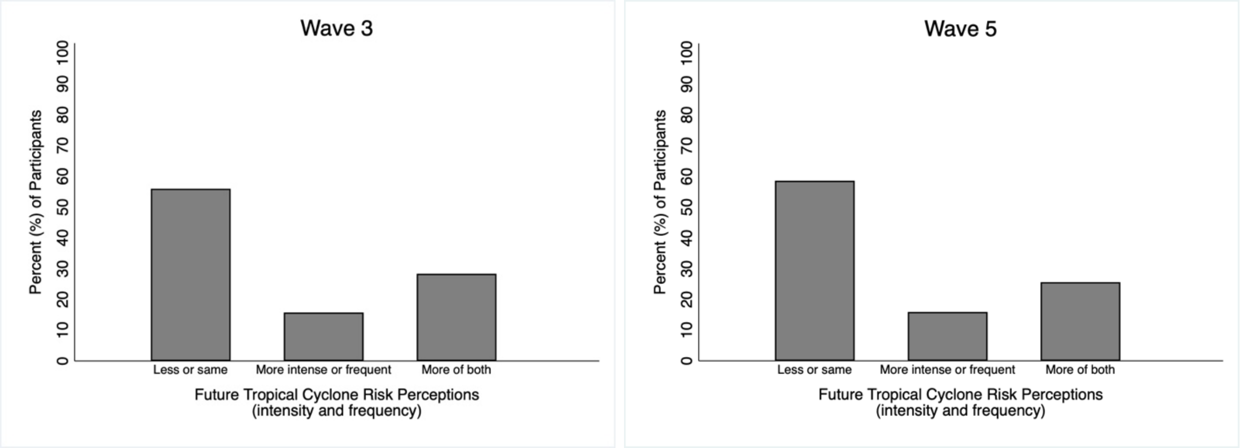

Histograms of future tropical cyclone risk perceptions at Waves 3 and 5. Each histogram is weighted to its own wave survey poststratification weights (e.g., Wave 3 includes all participants who completed up to Wave 3, Wave 5 illustrates data from participants who completed all waves).

Figure S5.

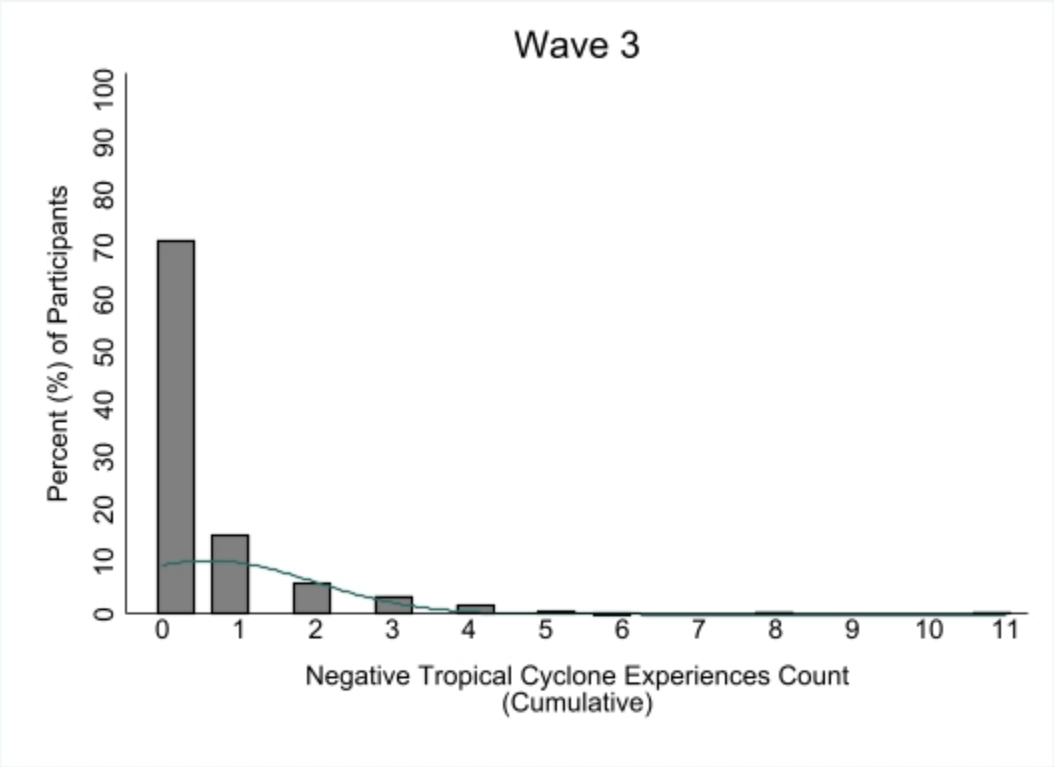

Histogram of cumulative self-reported negative tropical cyclone (hurricane) experiences summed across waves up to and including Wave 3. Wave 5 poststratification survey weights were applied. Line = normal density curve.

Figure S6.

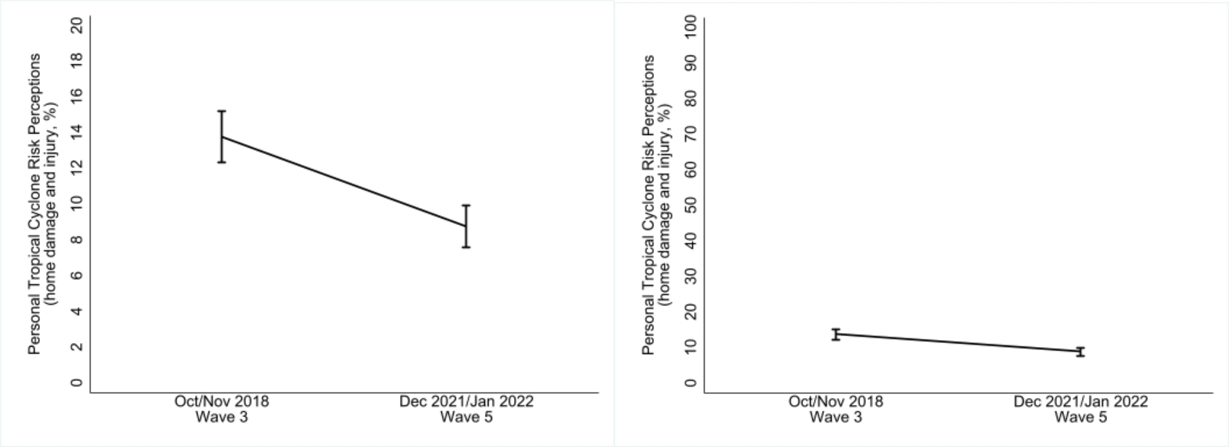

(A) Main effect of time on personal tropical cyclone risk perceptions between Waves 3 and 5, controlling for reported adaptation and covariates. Note the y-axis was rescaled for this illustration. (B) Main effect of time on personal tropical cyclone risk perceptions between Waves 3 and 5 illustrated to full scale, controlling for reported adaptations and covariates. Risk perceptions ranged from *0%* to *100%*. Analyses included Wave 5 poststratification survey weights and used unstructured covariance matrix.

Figure S7.

A

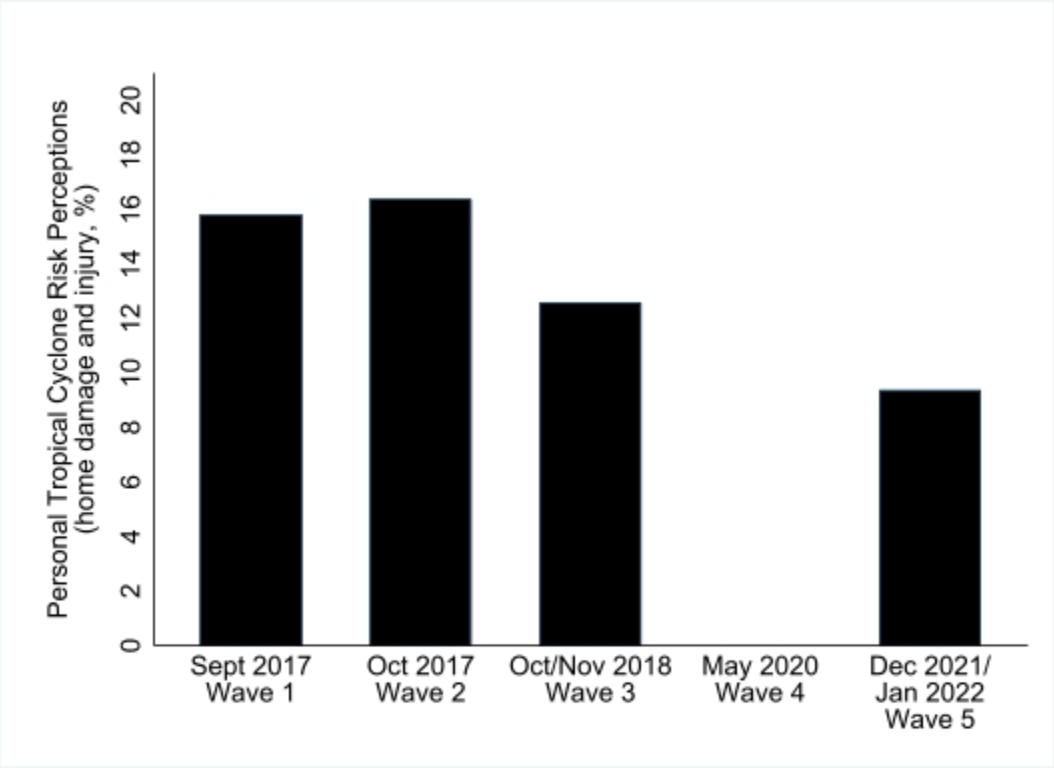

B

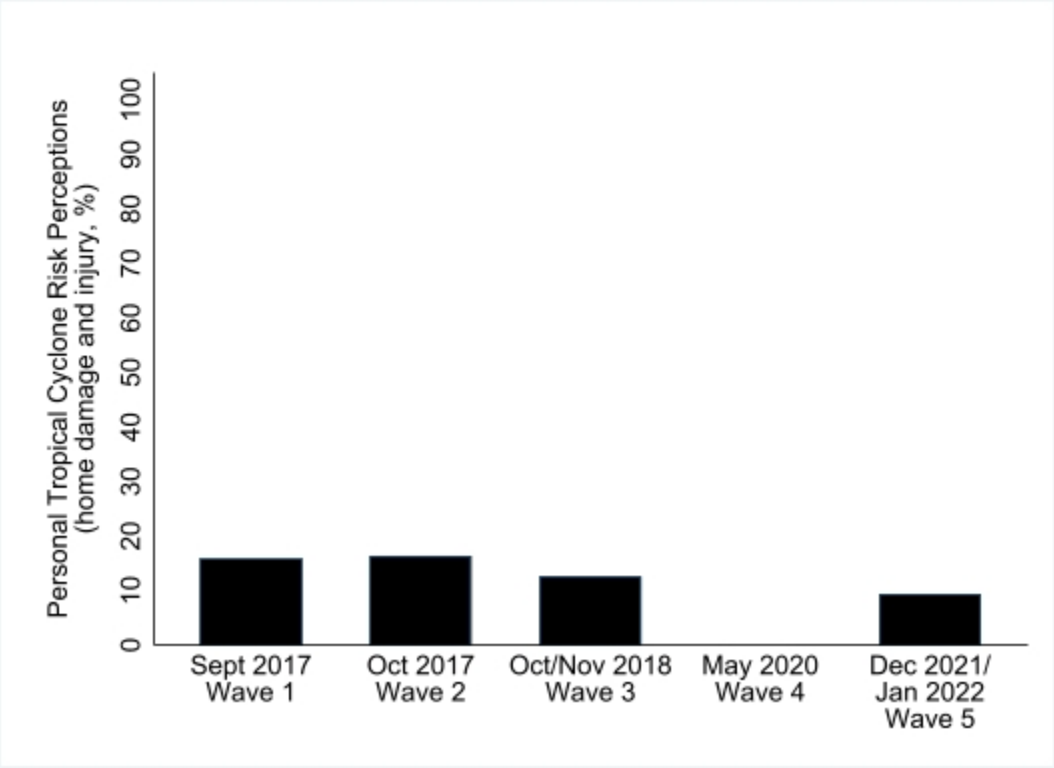

Average personal tropical cyclone (hurricane) risk perceptions at each wave illustrated to full scale. Wave 5 poststratification survey weights were applied.

Figure S8.

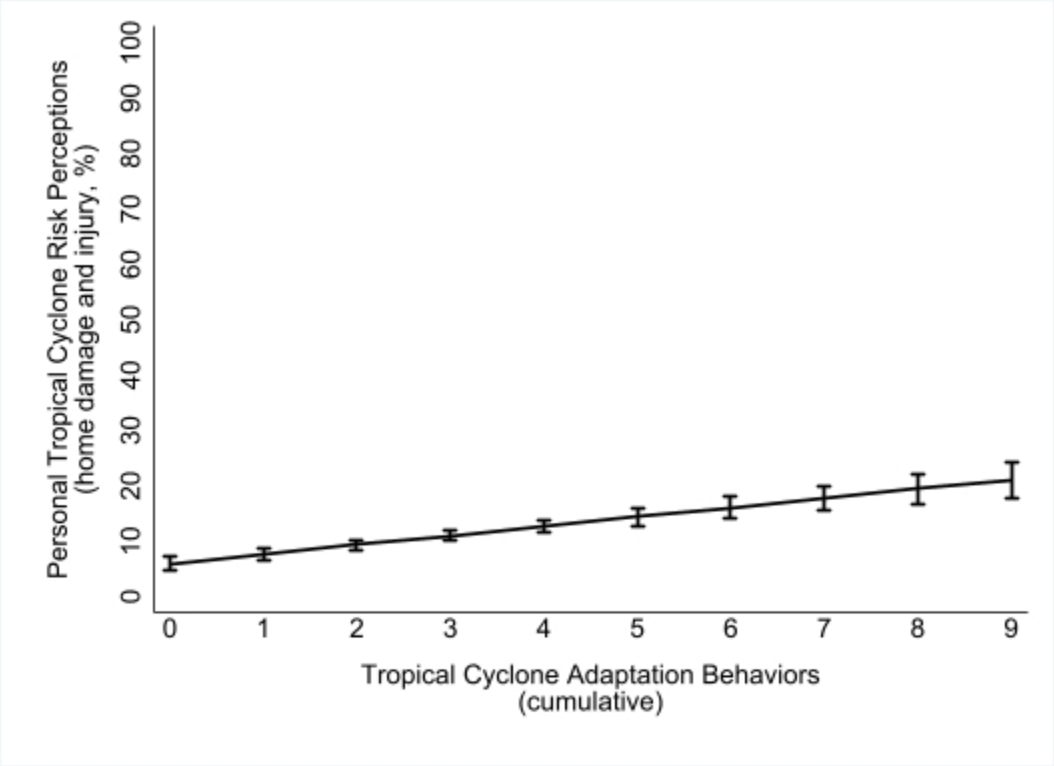

Main effect of cumulative self-reported adaptations count (Waves 3 through 5) on personal tropical cyclone risk perceptions across Waves 3 and 5 illustrated to full scale, controlling for time and covariates. Risk perceptions ranged from *0%* to *100%*. Wave 5 poststratification survey weights were applied. Bars represent 95% confidence intervals.

Figure S9.

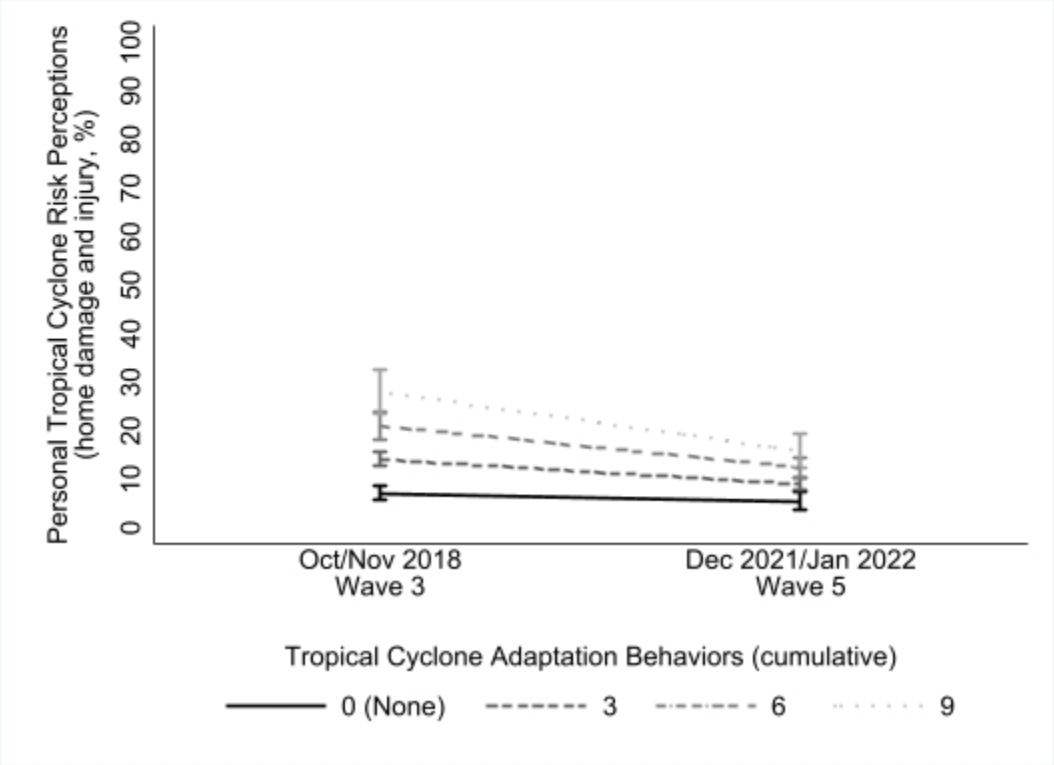

Interaction between time and reported tropical cyclone adaptations cumulative count on personal tropical cyclone risk perceptions between Waves 3 and 5, controlling for covariates, illustrated to full scale. Risk perceptions ranged from *0%* to *100%*. Wave 5 poststratification survey weights were applied. Bars represent 95% confidence intervals.

Figure S10.
A

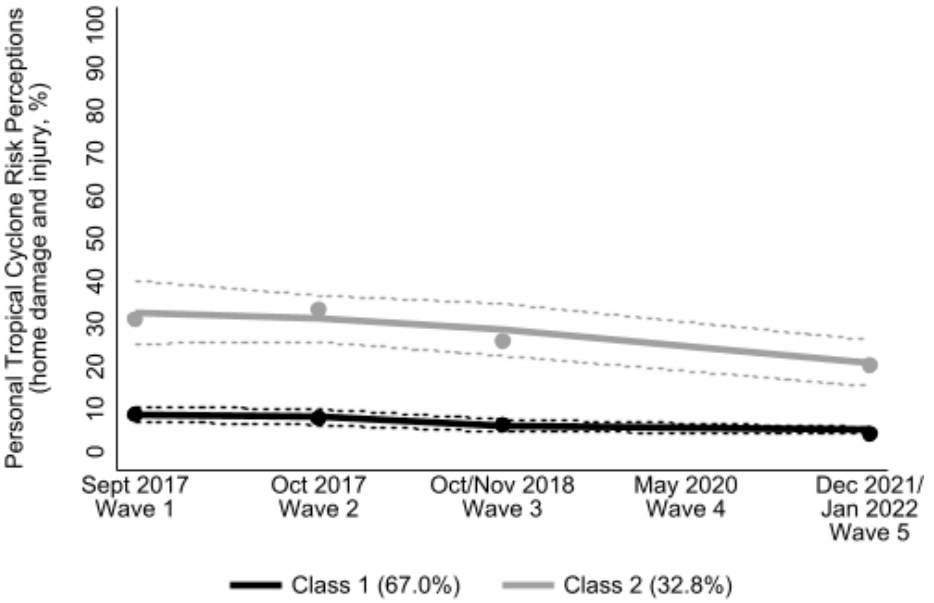

B

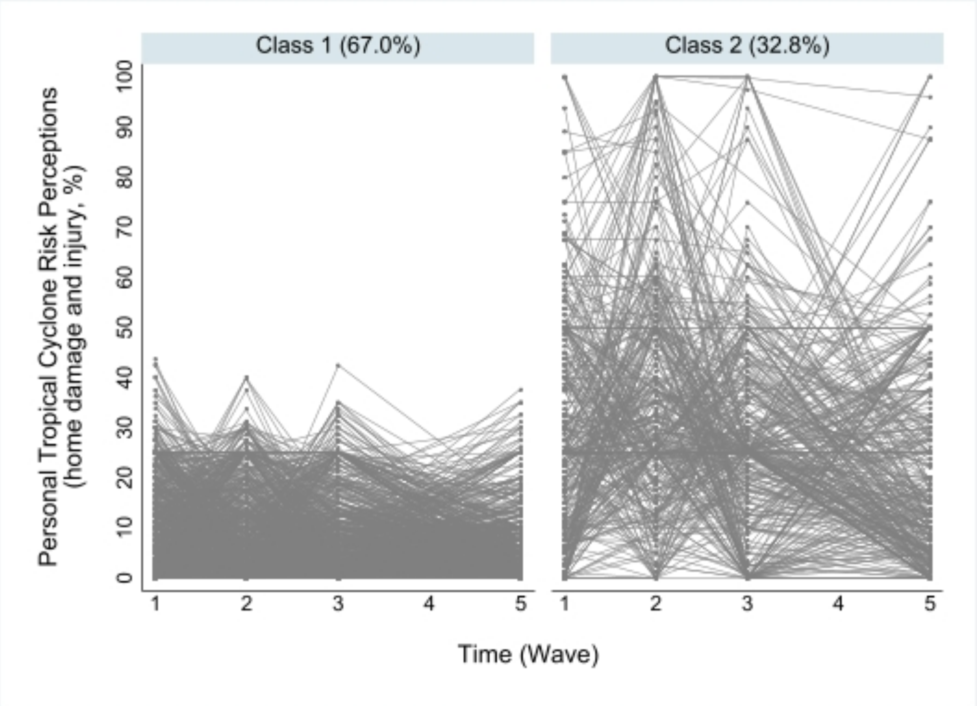

Results from group-based multi-trajectory (discrete/finite mixture) modeling predicting tropical cyclone risk perceptions over time with two latent classes of individuals: mean trajectories at full scale (A) and individual trajectories (B). (A) Dotted lines = bootstrapped 95% confidence intervals (10,000 repetitions). Wave 5 poststratification survey weights were applied and Wave 5 age, gender, race/ethnicity, education, income, state (TX/FL), and Wave 3 cumulative self-reported negative tropical cyclone (hurricane) experiences count were added as covariates.

Figure S11.

A

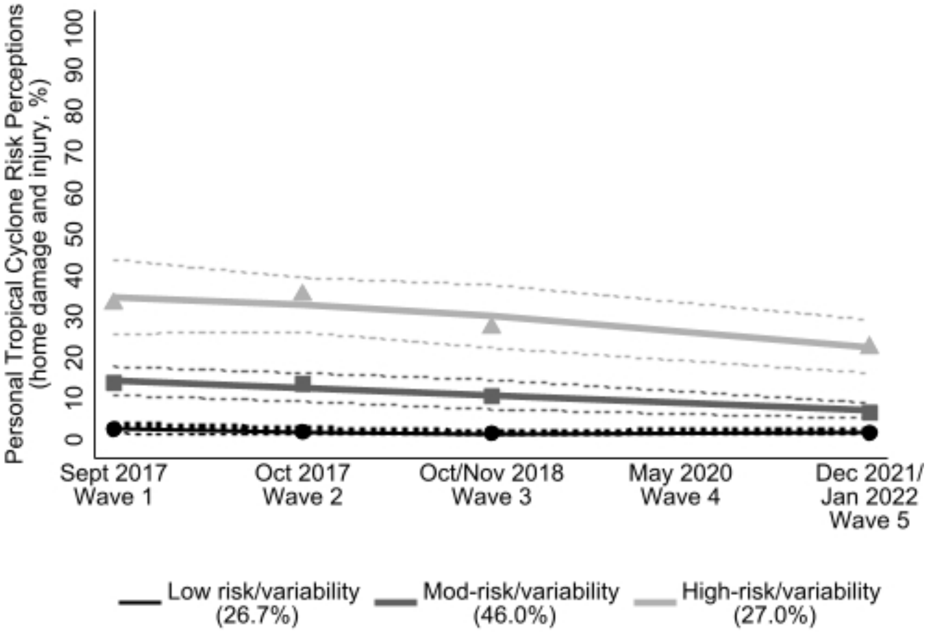

B

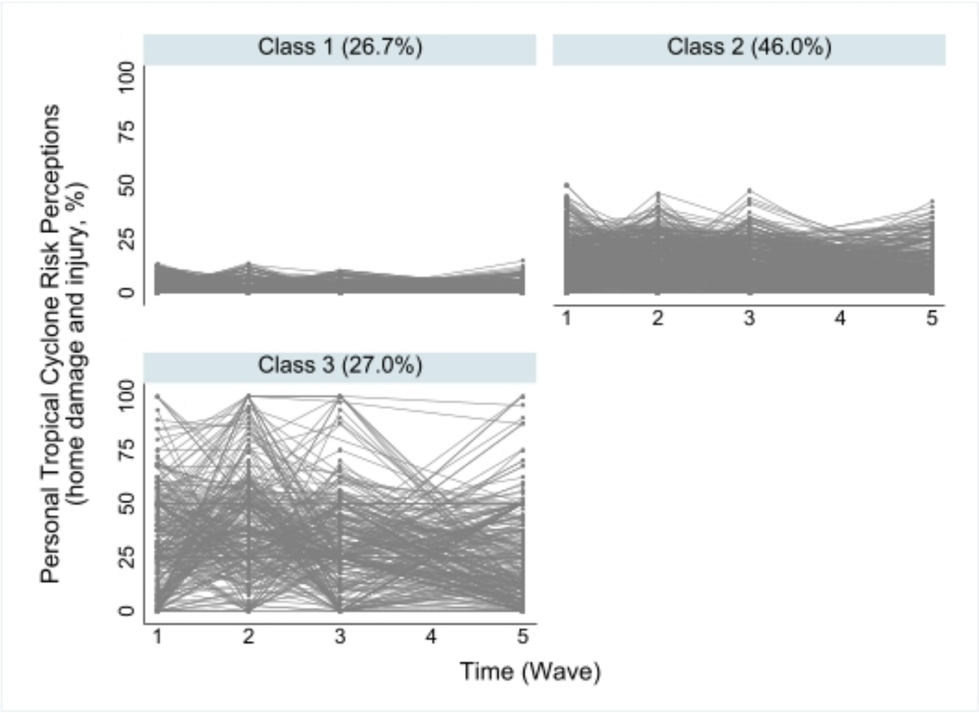

Results from group-based multi-trajectory (discrete/finite mixture) modeling predicting tropical cyclone risk perceptions over time with three latent classes of individuals: mean trajectories at full scale (A) and individual trajectories (B). (A) Dotted lines = bootstrapped 95% confidence

intervals (10,000 repetitions). Wave 5 poststratification survey weights were applied and t Wave 5 age, gender, race/ethnicity, education, income, state (TX/FL) and Wave 3 cumulative self-reported negative tropical cyclone (hurricane) experiences count were added as covariates.

Table S1.

Variable	Wave 1	Wave 2	Wave 3	Wave 4	Wave 5
Age – M(SD)	49.50(17.32)	49.72(17.20)	49.43(17.04)	51.00(16.75)	51.53(16.31)
Gender - n(%)					
Man	1,272(45.85)	1,176(45.85)	876(46.64)	864(46.81)	692(46.79)
Women	1,502(54.15)	1,388(54.15)	1,003(53.36)	982(53.19)	787(53.21)
Ethnicity - n(%)					
White Non-Hispanic	1,563(56.34)	1,462(57.01)	1,048(55.78)	1,011(54.76)	814(55.01)
Black Non-Hispanic	338(12.17)	295(11.49)	224(11.94)	220(11.91)	178(12.06)
Other Non-Hispanic	113(4.07)	100(3.90)	73(3.89)	68(3.70)	44(2.96)
Hispanic	724(26.10)	674(26.27)	510(27.15)	519(28.12)	408(27.60)
2+ Non-Hispanic	37(1.32)	34(1.33)	23(1.24)	28(1.50)	35(2.36)
Household income – M(SD)	11.93(4.68)	12.00(4.66)	11.80(4.84)	12.44(4.77)	12.69(4.72)
n(%)					
Under $10,000	184(6.64)	160(6.24)	147(7.81)	110(5.96)	74(5.00)
$10,000-$24,999	296(10.67)	276(10.77)	209(11.10)	187(10.12)	155(10.51)
$25,000-$49,999	661(23.83)	607(23.67)	445(23.70)	391(21.16)	309(20.87)
$50,000-$74,999	561(20.24)	516(20.14)	356(18.92)	363(19.66)	277(18.74)
$75,000-$99,999	420(15.12)	353(13.77)	290(15.41)	252(13.66)	204(13.81)
$100,000 +	652(23.51)	652(25.42)	433(23.06)	544(29.45	459(31.06)
Education - n(%)					
Less than high school	160(5.75)	146(5.71)	100(5.30)	82(4.44)	86(5.82)
High school	907(3.27)	834(32.52)	661(35.19)	630(34.13)	483(32.63)
Some college	919(33.13)	850(33.17)	613(32.61)	589(31.93)	456(30.83)
Bachelor's +	788(28.42)	733(28.60)	506(26.90)	545(29.50)	454(30.72)

Observed sociodemographic variables at surveyed waves (weighted at each wave). *Note.* M = mean, SD = standard deviation. Numbers may vary from total sample size due to missing data.

Table S2.

	Wave 1		Wave 2		Wave 3		Wave 4		Wave 5 n	
Complete		% %	n	%	n	%	n	%	n	
Yes	2,774	46.70	2,564	86.62	1,879	70.19	1,846	73.63	1,479	83.75
No	3,166	53.30	396	13.38	798	29.81	661	26.37	287	16.25
N	5,940	100.00	2,960	100.00	2,677	100.00	2,507	100.00	1,766	100.0

Weighted survey completion for participants by wave. *Note.* All participants who had completed Wave 1 were invited to complete each of the subsequent waves. *N* = total invited to participate and decreases over time due to attrition from the KnowledgePanel; withdraw panelists who agreed to be contacted for future waves were invited to complete subsequent surveys.

Table S3.

**Variables**		**Wave 1**	**Wave 2**	**Wave 3**	**Wave 4**	**Wave 5**
		n (%)	n (%)	n (%)	n (%)	n (%)
**Personal Adaptation** (cumulative)		--	--	n = 1,879	n = 1,846	n = 1,479
Mean (SD)		--	--	2.30(2.31)	3.15(2.33)	3.62 (2.37)
Cronbach's α		--	--	.784	.836	.842
0 = no adaptation		--	--	626(33.31)	283(16.17)	189(13.14)
1		--	--	244(13.00)	239(13.64)	157(10.88)
2		--	--	236(12.57)	240(13.71)	163(11.32)
3		--	--	253(13.45)	240(13.70)	149(10.35)
4		--	--	170(9.07)	238(13.61)	230(16.00)
5		--	--	122(6.47))	178(10.14)	198(13.76)
6		--	--	100(5.31)	162(9.25)	165(11.44)
7		--	--	72(3.83)	105(6.01)	125(8.69)
8		--	--	54(2.88)	63(3.62)	61(4.24)
9 = nine adaptation behaviors		--	--	2(0.10)	2(0.14)	3(0.19)
Learn about the risks from hurricanes and how to prepare for them	No	--	--	1,101(58.61)	715(42.57)	494(35.44)
	Yes	--	--	778(41.39)	964(57.43)	901(64.56)
Put together an emergency kit (e.g., food, medical supplies, flashlight)	No	--	--	900(47.89)	514(30.34)	343(24.14)
	Yes	--	--	979(52.11)	1,181(69.66)	1,078(75.86)
Develop and practice an emergency plan	No	--	--	1,453(77.35)	1,051(65.08)	798(59.51)
	Yes	--	--	426(22.65)	564(34.92)	543(40.49)
Identify shelter locations in the event of an evacuation	No	--	--	1,396(74.29)	953(58.17)	697(51.70)
	Yes	--	--	483(25.71)	685(41.83)	651(48.30)
Copy important documents (e.g., birth certificates, licenses)	No	--	--	1,336(71.08)	892(54.41)	652(48.12)
	Yes	--	--	544(28.92)	747(45.59)	703(51.88)
Get a rowboat or inflatable raft	No	--	--	1,799(95.76)	1,460(93.82)	1,193(93.52)
	Yes	--	--	80(4.24)	96(6.18)	83(6.48)
Make my home more hurricane proof (e.g., install hurricane shutters, sandbags)	No	--	--	1,329(70.71)	944(58.50)	688(51.57)
	Yes	--	--	550(29.29)	669(41.50)	646(48.43)
Have flood insurance	No	--	--	1,463(77.85)	1,088(67.67)	824(61.81)
	Yes	--	--	416(22.15)	520(32.33)	509(38.19)
Other (please specify)	No	--	--	1,817(96.70)	1,474(94.36)	1,182(92.04)
	Yes	--	--	62(3.30)	88(5.64)	102(7.96)

Weighted descriptive statistics for cumulative Personal Adaptation at Waves 3-5, including individual items (weighted at each wave). SD = standard deviation. Numbers may vary from total sample size due to missing data.

Table S4.

Variables		Wave 1	Wave 2	Wave 3	Wave 4	Wave 5
		n (%)	n (%)	n (%)	n (%)	n (%)
**Personal Adaptation** (non-cumulative)		--	--	n = 1,879	n = 1,846	n = 1,479
Mean (SD)		--	--	2.30(2.31)	2.02(2.01)	1.92(1.90)
Cronbach's α		--	--	.784	.791	.782
0 = no adaptation		--	--	626(33.31)	584(31.84)	482(32.59)
1		--	--	244(13.00)	336(18.30)	274(18.54)
2		--	--	236(12.57)	270(14.74)	203(13.75)
3		--	--	253(13.45)	208(11.31)	198(13.40)
4		--	--	170(9.07)	173(9.44)	160(10.82)
5		--	--	122(6.47))	135(7.33)	82(5.55)
6		--	--	100(5.31)	87(4.74)	50(3.37)
7		--	--	72(3.83)	26(1.44)	25(1.69)
8		--	--	54(2.88)	16(0.85)	4(0.26)
9 = nine adaptation behaviors		--	--	2(0.10)	1(0.02)	1(0.02)
Learn about the risks from hurricanes and how to prepare for them	No	--	--	1,101(58.61)	1,164(63.46)	934(63.65)
	Yes	--	--	778(41.39)	670(36.54)	533(36.35)
Put together an emergency kit (e.g., food, medical supplies, flashlight)	No	--	--	900(47.89)	953(51.97)	702(47.84)
	Yes	--	--	979(52.11)	881(48.03)	765(52.16)
Develop and practice an emergency plan	No	--	--	1,453(77.35)	1,487(81.03)	1,275(86.92)
	Yes	--	--	426(22.65)	348(18..97)	192(13.08)
Identify shelter locations in the event of an evacuation	No	--	--	1,396(74.29)	1,83(75.39)	1,160(79.07)
	Yes	--	--	483(25.71)	451(24.61)	307(20.93)
Copy important documents (e.g., birth certificates, licenses)	No	--	--	1,336(71.08)	1,310(71.40)	1,100(75.01)
	Yes	--	--	544(28.92)	525(28.60)	367(24.99)
Get a rowboat or inflatable raft	No	--	--	1,799(95.76)	1,794(97.78)	1,441(98.25)
	Yes	--	--	80(4.24)	41(2.22)	26(1.75)
Make my home more hurricane proof (e.g., install hurricane shutters, sandbags)	No	--	--	1,329(70.71)	1,431(78.01)	1,136(77.46)
	Yes	--	--	550(29.29)	403(21.99)	331(22.54)
Have flood insurance	No	--	--	1,463(77.85)	1,501(81.84)	1,199(81.71)
	Yes	--	--	n = 1,879	n = 1,846	268(18.29)
Other (please specify)	No	--	--	2.30(2.31)	2.02(2.01)	1,422(96.94)
	Yes	--	--	.784	.791	45(3.06)

Weighted descriptive statistics for non-cumulative self-reported tropical cyclone (hurricane) adaptations at Waves 3-5, including individual items (weighted at each wave). SD = standard deviation. Numbers may vary from total sample size due to missing data.

Table S5.

Personal Risk Perceptions	Wave 1<br>n = 2,774	Wave 2<br>n = 2,564	Wave 3<br>n = 1,879	Wave 4	Wave 5<br>n = 1,479
		Mean (Standard Deviation)			
Overall Composite	15.64 (17.67)	15.71 (18.95)	12.86 (17.49)	--	9.38 (14.93)
Cronbach's α	.768	.804	.835	--	.879
Pearson r (p)	.462 (< .001)	.537 (< .001)	.561 (< .001)	--	.695 (< .001)
Home Damage					
Average Composite	16.88 (21.33)	17.13 (22.70)	14.91 (21.48)	--	11.29 (18.51)
Cronbach's α	.686	.796	.772	--	.804
Pearson r (p)	.563 (< .001)	.672 (< .001)	.648 (< .001)	--	.688 (< .001)
Your home will be severely damaged or destroyed because of a hurricane or its aftermath	20.98 (25.12)	22.14 (27.38)	17.21 (24.36)	--	12.88 (20.21)
You will never be able to return to your current home as a result of a hurricane or its aftermath	12.51 (23.18)	11.95 (22.06)	12.38 (22.92)	--	9.57 (19.89)
Injury					
Average Composite	14.74 (20.02)	14.41 (20.71)	10.93 (17.57)	--	8.01 (15.44)
Cronbach's α	.692	.699	.787	--	.874
Pearson r (p)	.505 (< .001)	.543 (< .001)	.608 (< .001)	--	.730 (< .001)
You will be seriously injured by a hurricane or its aftermath	9.01 (17.30)	8.59 (17.60)	8.08 (16.00)	--	6.40 (13.89)
Someone close to you will be seriously injured by a hurricane or its aftermath	20.38 (27.69)	19.95 (28.48)	13.70 (22.41)	--	9.17 (17.16)

Weighted descriptive statistics for personal tropical cyclone risk perceptions at Waves 1-3 and 5, including individual items (weighted at each wave). Overall personal risk perceptions composite is an average of home damage and injury risk perceptions, which are each averages of two items. Range = 0-100.

Table S6.

Future Tropical Cyclone Risk Perceptions	Wave 1<br>n (%)	Wave 2<br>n (%)	Wave 3<br>n (%)	Wave 4<br>n (%)	Wave 5<br>n (%)
N	--	--	1,879	--	1,479
Mean (SD)	--	--	0.71 (0.87)	--	0.67 (0.86)
Cronbach's α	--	--	.804	--	.790
Pearson r correlation	--	--	.680 (< .001)	--	.679 (< .001)
0 = less or about the same intensity/frequency	--	--	1,048 (56.53)	--	864 (58.54)
1 = either more intensity or frequency	--	--	290 (15.62)	--	233 (15.79)
2 = more intensity or frequency	--	--	517 (27.87)	--	379 (25.67)
Do you think hurricanes during future hurricane seasons will be?					
Less intense	--	--	53 (2.85)	--	60 (4.07)
About the same	--	--	1,075 (58.12)	--	904 (61.55)
More intense	--	--	722 (39.03)	--	505 (34.38)
Do you think hurricanes during future hurricane seasons will be?					
Less frequent	--	--	79 (4.28)	--	67 (4.60)
About the same	--	--	1,164 (63.12)	--	915 (62.34)
More frequent	--	--	601 (32.60)	--	485 (33.07)

Weighted descriptive statistics for future tropical cyclone risk perceptions at Waves 3 and 5, including individual items (weighted at each wave). SD = standard deviation. Numbers may vary from total sample size due to missing data.

Table S7.

Variables		Wave 1 n (%)	Wave 2 n (%)	Wave 3 n (%)	Wave 4 n (%)	Wave 5 n (%)
**Lifetime Negative Tropical Cyclone Experiences** (cumulative)		n = 2,774	n = 2,564	n = 1,879	--	--
Mean (SD)				0.50 (1.10)	--	--
Cronbach's α				.728	--	--
0 = no experience				1,346 (71.75)	--	--
1				315 (16.77)	--	--
2				121 (6.44)	--	--
3				54 (0.29)	--	--
4				16 (0.01)	--	--
5				11 (0.01)	--	--
6				3 (0.002)	--	--
7				1(< .001)	--	--
8				7(0.01)	--	--
9				0(0.00)	--	--
10				0(0.00)	--	--
11				3(0.002)	--	--
I lost property in the hurricane or its aftermath	No	2,445 (90.32)	2,036 (83.03)	791 (72.36)	--	--
	Yes	262 (9.68)	416 (16.97)	302 (27.64)	--	--
My home was totally destroyed in the hurricane or its aftermath	No	2,678 (98.95)	2,387 (98.10)	985 (96.15)	--	--
	Yes	28 (1.05)	46 (1.90)	39 (3.85)	--	--
I was injured in the hurricane or its aftermath	No	2,693 (99.51)	2,408 (98.97)	999 (97.15)	--	--
	Yes	13 (0.49)	25 (1.03)	29 (2.85)	--	--
I lost a pet in the hurricane or its aftermath	No	2,693 (99.48)	2,397 (98.36)	994 (96.55)	--	--
	Yes	14 (0.52)	40 (1.64)	36 (3.45)	--	--
I knew / know someone who was injured in the hurricane or its aftermath	No	2,434 (89.94)	2,100 (85.72)	1,436 (82.81)	--	--
	Yes	272 (10.06)	350 (14.28)	298 (17.19)	--	--
I knew someone who was killed in the hurricane or its aftermath	No	2,658 (98.19)	2,356 (96.49)	1,652 (95.99)	--	--
	Yes	49 (1.81)	86 (3.51)	69 (4.01)	--	--

Weighted descriptive statistics for cumulative self-reported Lifetime Negative [Tropical Cyclone] Experiences at Waves 1-3, including individual items (weighted at each wave). SD = standard deviation. Numbers may vary from total sample size due to missing data.

Table S8.

Polynomial Function (# of Classes)	AIC	Individual BIC (N = 1476)	Observation BIC (N = 5531)	Entropy
Linear				
2	-18259.89	-18296.97	-18306.22	.779
3	-18019.65	-18085.86	-18102.38	.714
4	-17857.81	-17953.16	-17976.94	.729
Quadratic				
2	-18260.02	-18302.40	-18312.97	.779
3	-18015.03	-18089.19	-18107.69	.716
4	-17852.71	-17958.65	-17985.08	.731
Cubic				
2	-18251.87	-18299.54	-18311.43	.778
3	-18007.04	-18089.14	-18109.62	.717
4	-17843.83	-17960.37	-17989.43	.734

Comparison of personal tropical cyclone risk perceptions (Waves 1-3, 5) group-based trajectory models to determine the number of latent classes. *Note.* Each latent class had the same polynomial function within each polynomial function type (e.g., all groupings for "Quadratic" had quadratic polynomials); AIC = Akaike's Information Criterion; BIC = Bayesian Information Criterion (lower absolute value = better model fit); Entropy higher values = better latent class delineation; Each model controlled for Wave 5 age, gender, race/ethnicity, education, income, state (TX/FL), and cumulative self-reported negative tropical cyclone (hurricane) experience count (Wave 3), and included Wave 5 poststratification survey weights; The four-class solutions produced two latent class trajectories that mostly overlapped, making interpretability difficult; Wave 5 poststratification survey weights were applied. N's may differ from total sample size due to missing data.

Table S9.

Polynomial Function	AIC	Individual BIC (N = 1476)	Observation BIC (N = 5531)	Entropy
2 Latent Classes				
1 1	-18259.89	-18296.97	-18306.22	.779
1 2	-18259.61	-18299.34	-18309.24	.779
1 3	-18252.60	-18294.97	-18305.54	.778
2 1	-18260.46	-18300.18	-18310.09	.779
2 2	-18260.02	-18302.40	-18312.97	.779
2 3	-18253.08	-18298.10	-18309.33	.778
3 1	-18258.78	-18301.16	-18311.72	.780
3 2	-18258.24	-18303.26	-18314.49	.780
3 3	-18251.87	-18299.54	-18311.43	.778
3 Latent Classes				
1 1 1	-18019.65	-18085.86	-18102.38	.714
1 1 2	-18019.98	-18088.84	-18106.01	.714
1 1 3	-18015.06	-18086.57	-18104.41	.713
1 2 1	-18020.15	-18089.01	-18106.19	.715
1 2 2	-18020.60	-18092.11	-18109.95	.715
1 2 3	-18015.63	-18089.79	-18108.28	.713
1 3 1	-18015.30	-18086.81	-18104.65	.717
1 3 2	-18015.67	-18089.83	-18108.33	.717
1 3 3	-18011.70	-18088.50	-18107.66	.715
2 1 1	-18014.44	-18083.30	-18100.48	.716
2 1 2	-18014.74	-18086.25	-18104.09	.715
2 1 3	-18009.78	-18083.94	-18102.44	.714
2 2 1	-18014.56	-18086.07	-18103.91	.716
2 2 2	-18015.03	-18089.19	-18107.69	.716
2 2 3	-18009.99	-18086.80	-18105.95	.714
2 3 1	-18009.81	-18083.97	-18102.46	.718
2 3 2	-18010.20	-18087.01	-18106.16	.718
2 3 3	-18006.15	-18085.60	-18105.42	.717
3 1 1	-18015.11	-18086.62	-18104.45	.716
3 1 2	-18015.41	-18089.57	-18108.07	.715
3 1 3	-18010.46	-18087.27	-18106.43	.714

3 2 1	-18015.20	-18289.36	-18107.86	.716
3 2 2	-18015.68	-18092.48	-18111.64	.716
3 2 3	-18010.64	-18090.10	-18109.91	.715
3 3 1	-18010.70	-18087.51	-18106.66	.718
3 3 2	-18011.09	-18090.55	-18110.37	.718
3 3 3	-18007.04	-18089.14	-18109.62	.717

Comparison of personal tropical cyclone risk perceptions (Waves 1-3, 5) group-based trajectory (discrete/finite mixture) models to determine the order of polynomial functions. *Note.* AIC = Akaike's Information Criterion; BIC = Bayesian Information Criterion (lower = better model fit); Entropy higher values = better latent class delineation; Bold = best fitting model per number of classes; Bold and italicized = chosen final model based on visual inspection comparing the two best fitting model with three classes. Each model controlled for Wave 5 age, gender, race/ethnicity, education, and income and included Wave 5 poststratification survey weights.

Table S10.

Variable	(1)	(2)	(3)	(4)	(5)	(6)	(7)
(1) Personal risk perceptions	1.00						
(2) Future risk perceptions	.14<br>< .001	1.00					
(3) Adaptations (cumulative)	.21<br>< .001	.08<br>< .001	1.00				
(4) Negative TC experience (cumulative to Wave 3)	.19<br>< .001	.07<br>.002	.19<br>< .001	1.00			
(5) Age	-.07<br>< .001	-.07<br>< .001	.12<br>< .001	-.08<br>< .001	1.00		
(6) Household income	-.14<br>< .001	-.03<br>1.00	-.001<br>1.00	-.03<br>.011	-.003<br>1.00	1.00	
(7) Education	-.10<br>< .001	.07<br>.001	-.01<br>1.00	.04<br>.001	-.07<br>< .001	.33<br>< .001	1.00

Unweighted zero-order Pearson *r* correlations across waves. *Note.* First row of each cell = Pearson *r* correlation coefficient, second row of each cell = *p*-value with Bonferroni-adjusted α = .05 / 21 = .002. Personal tropical cyclone risk perceptions = % chance of home damage and personal or close other injury risks. Future tropical cyclone risk perception = intensity and frequency (0 = *less or same*, 1 = *more intense or frequent*, 2 = *more intense and frequent*). TC = tropical cyclone. Self-reported negative tropical cyclone experiences is a total count across Waves 1-3 summed at Wave 3. Income ranged from 1 (*less than $5,000)* to 21 (*$250,000 or more*) and education ranged from 1 (*less than high school*) to 4 (*Bachelor's degree or higher*).

Table S11.

Variable	Coeff.	SE_{Robust}	p	95% CI LL	95% CI UL
Main predictors					
Time	-2.50	0.43	< .001	-3.35	-1.65
Adaptation	1.68	0.23	< .001	1.23	2.13
Covariates					
Age	-0.02	0.03	.569	-0.08	0.04
Gender (ref = man)					
Woman	2.42	0.93	.009	0.59	4.25
Ethnicity (ref = White, non-Hispanic)					
Black, non-Hispanic	1.44	1.75	.410	-1.99	4.87
Other, non-Hispanic	-1.63	2.63	.534	-6.78	3.52
Hispanic	1.79	1.33	.177	-0.81	4.39
2+, non-Hispanic	3.25	3.01	.279	-2.64	9.14
Household income	-0.11	0.11	.316	-0.33	0.11
Education (ref = no college degree)					
College degree	-1.37	1.01	.177	-3.35	0.62
State (ref = Texas)					
Florida	2.49	1.01	.014	0.51	4.47
Intercept	15.41	3.16	< .001	9.22	21.60
Random effects parameters					
Participant					
Adaptation variance	2.09	0.98		0.83	5.26
Intercept variance	9.03	7.10		1.93	42.18
Adaptation - intercept covariance	4.35	1.21		1.98	6.71
Residual variance	158.28	17.32		127.73	196.14
Model fit	$\chi^2(11) = 152.53$, $p < .001$, AIC = 21548.97, BIC = 21633.25				

Linear mixed effects model predicting personal tropical cyclone risk perceptions (%) from time, cumulative self-reported tropical cyclone household adaptation count, and covariates (main effects). *Note.* Coeff. = unstandardized coefficient; CI = confidence intervals; LL = lower limit; UL = upper limit; Ref = reference comparison group; AIC = Akaike's Information Criterion; BIC = Bayesian Information Criterion; Personal tropical cyclone risk perceptions (home damage and injury composite) ranged from *0%* to *100%*; Models were estimated using Wave 5 poststratification survey weights, maximum likelihood estimation, and an unstructured covariance matrix.

Table S12.

Variable	Coeff.	SE_{Robust}	p	95% CI LL	95% CI UL
Main predictors					
Time	-0.79	0.49	.106	-1.75	0.17
Adaptation	4.08	0.72	< .001	2.67	5.49
Time X Adaptation	-0.59	0.16	< .001	-0.90	-0.27
Covariates					
Age	-0.02	0.03	.537	-0.08	0.04
Gender (ref = man)					
Woman	2.50	0.93	.007	0.67	4.33
Ethnicity (ref = White, non-Hispanic)					
Black, non-Hispanic	1.57	1.76	.370	-1.87	5.02
Other, non-Hispanic	-1.88	2.72	.489	-7.21	3.45
Hispanic	1.77	1.33	.183	-0.83	4.38
2+, non-Hispanic	3.22	2.97	.279	-2.60	9.04
Household income	-0.11	0.11	.321	-0.33	0.11
Education (ref = no college degree)					
College degree	-1.41	1.01	.161	-3.39	0.56
State (ref = Texas)					
Florida	2.53	1.01	.013	0.54	4.51
Intercept	8.80	3.05	< .001	2.83	14.77
Random effects parameters					
Participant					
Adaptation variance	2.12	1.00		0.84	5.36
Intercept variance	9.74	7.58		2.12	44.80
Adaptation - intercept covariance	4.55	1.21		2.17	6.92
Residual variance	154.51	16.83		124.82	191.27
Model fit	$\chi^2(12) = 149.90$, $p < .001$, AIC = 21521.02, BIC = 21610.57				

Linear mixed effects model predicting personal tropical cyclone risk perceptions (%) from time, cumulative self-reported tropical cyclone household adaptation count, time X adaptation count interaction, and covariates. *Note.* Coeff. = unstandardized coefficient; CI = confidence intervals; LL = lower limit; UL = upper limit; Ref = reference comparison group; AIC = Akaike's Information Criterion; BIC = Bayesian Information Criterion; Personal tropical cyclone risk perceptions (home damage and injury composite) ranged from *0%* to *100%*; Models were estimated using Wave 5 poststratification survey weights, maximum likelihood estimation, and an unstructured covariance matrix.

Table S13.

Class	Parameter	Estimate	*SE*	*p*
1 (*n* = 1103)	Intercept	2.94	3.67	.423
	Linear	7.08	5.59	.206
	Quadratic	-3.76	2.33	.108
	Cubic	0.45	0.27	.101
2 (*n* = 373)	Intercept	32.08	5.57	< .001
	Linear	0.14	4.05	.973
	Quadratic	-0.62	0.63	.325
1	Sigma	11.79	0.55	< .001
	APP	.9483		
	OCC	6.76		
	Prob	.7473		
	Total prob	.7306		
2	Sigma	24.34	1.32	< .001
	APP	.9131		
	OCC	28.51		
	Prob	.2527		
	Total prob	.2694		
Group Membership				
1	Baseline			
2	Constant	-0.50	0.68	.462
	Age	-0.01	0.01	.104
	Gender	0.60	0.27	.026
	Ethnicity	0.01	0.10	.939
	Education	-0.79	0.28	.005
	Income	-0.06	0.03	.067
	State	0.88	0.26	.001
	Negative TC experiences	0.98	0.13	< .001

Model statistics

Akaike's Information Criterion = -18258.24

Bayesian Information Criterion (*N* = 1476) = -18303.26

Entropy = .780

Group-based multi-trajectory (discrete/finite mixture) modeling results for personal tropical cyclone risk perceptions (%) over time with two latent classes. *Note.* APP = average posterior probability; OCC = weighted posterior proportions for the odds of correct classification; Prob = proportion in each class based on the assignments for the maximum posterior probability; Total prob = expected number in each class based on the sums of the posterior probabilities. Parameters were estimated using Wheeler's code (*7*); Wave 5 age, gender (male = 0, female = 1), race/ethnicity, education, income, state (Texas = 0, Florida = 1), and Wave 3 negative tropical cyclone (TC) experiences total count were covariates. Education = 0 (no college degree), 1 (college degree or higher); Covariate estimates under "Group Membership" are the log-odds estimates for the risk factors for each group relative to group (latent class) 1. Wave 5 poststratification survey weights were applied.

Table S14.

Class	Parameter	Estimate	*SE*	*p*
1 (*n* = 497)	Intercept	1.23	3.74	.742
	Linear	0.37	5.51	.946
	Quadratic	-0.84	2.30	.717
	Cubic	0.14	0.27	.606
2 (*n* = 707)	Intercept	14.87	2.29	< .001
	Linear	-1.24	1.81	.493
	Quadratic	-0.19	0.29	.501
3 (*n* = 272)	Intercept	35.03	6.81	< .001
	Linear	-1.00	5.03	.842
	Quadratic	-0.44	0.78	.574
1	Sigma	5.24	0.74	< .001
	APP	.8711		
	OCC	14.67		
	Prob	.3367		
	Total prob	.3153		
2	Sigma	12.17	0.68	< .001
	APP	.8799		
	OCC	8.01		
	Prob	.4790		
	Total prob	.4777		
3	Sigma	26.26	1.63	< .001
	APP	.9223		
	OCC	45.45		
	Prob	.1843		
	Total prob	.2070		
		Group Membership		
1	Baseline			
2	Constant	-0.21	1.02	.834
	Age	-0.001	0.01	.913
	Gender	-0.15	0.26	.574
	Ethnicity	0.12	0.12	.334
	Education	-0.43	0.25	.088
	Income	0.02	0.04	.558
	State	0.78	0.29	.007
	Negative TC experiences	0.39	0.19	.042
3	Constant	0.16	0.88	.856
	Age	-0.02	0.01	.064
	Gender	0.60	0.33	.072
	Ethnicity	0.05	0.13	.695
	Education	-1.22	0.35	< .001
	Income	-0.06	0.04	.125
	State	1.29	0.32	< .001
	Negative TC experiences	1.21	0.19	< .001

Group-based multi-trajectory (discrete/finite mixture) modeling results for personal tropical cyclone risk perceptions (%) over time with three latent classes. *Note.* APP = average posterior probability; OCC = weighted posterior proportions for the odds of correct classification; Prob = proportion in each class based on the assignments for the maximum posterior probability; Total prob = expected number in each class based on the sums of the posterior probabilities. Parameters were estimated using Wheeler's code (*7*); Wave 5 age, gender (male = 0, female = 1), race/ethnicity, education, income, state (Texas = 0, Florida = 1), and Wave 3 negative tropical cyclone (TC) experiences total count were covariates. Education = 0 (no college degree), 1 (college degree or higher); Covariate estimates under "Group Membership" are the log-odds estimates for the risk factors for each group relative to group (latent class) 1. Wave 5 poststratification survey weights were applied.

Table S15.

Variable	Coefficient	SE_{Robust}	p	95% CI LL	95% CI UL
Main predictors					
Time	-1.70	0.19	< .001	-2.08	-1.32
Negative tropical cyclone experiences count	3.78	0.66	< .001	2.48	5.07
Covariates					
Age	-0.03	0.04	.426	-0.10	0.04
Gender (ref = man)					
Woman	2.74	1.00	.006	0.78	4.70
Ethnicity (ref = White, non-Hispanic)					
Black, non-Hispanic	1.26	1.72	.464	-2.11	4.63
Other, non-Hispanic	-2.33	2.05	.255	-6.35	1.68
Hispanic	1.32	1.39	.340	-1.40	4.05
2+, non-Hispanic	0.32	1.96	.869	-3.51	4.16
Household income	-0.18	0.12	.150	-0.42	0.06
Education (ref = no college degree)					
College degree	-2.89	1.01	.004	-4.87	-0.92
State (ref = Texas)					
Florida	3.15	1.06	.003	1.07	5.23
Intercept	17.09	2.79	< .001	11.62	22.55
Random effects parameters					
Participant					
Negative tropical cyclone experiences variance	6.39	6.18		0.96	42.54
Intercept variance	94.48	17.84		65.26	136.79
Negative tropical cyclone experiences - intercept covariance	-1.94	14.74		-30.83	26.94
Residual variance	175.44	12.48		152.60	201.69
Model fit	$\chi^2(11) = 175.06$, $p < .001$, AIC = 45462.82, BIC = 45547.58				

Linear mixed effects model predicting personal tropical cyclone risk perceptions (%) from time, self-reported negative tropical cyclone experiences total count (cumulative up to Wave 3), and covariates (main effects). *Note.* CI = confidence intervals; LL = lower limit; UL = upper limit; Ref = reference comparison group; AIC = Akaike's Information Criterion; BIC = Bayesian Information Criterion; Personal tropical cyclone risk perceptions (home damage and injury composite) ranged from *0%* to *100%*; Models were estimated using Wave 5 poststratification survey weights, maximum likelihood estimation, and an unstructured covariance matrix.

a
Table S16.

Variable	Coefficient	SE_{Robust}	p	95% CI LL	95% CI UL
Main predictors					
Time	-1.52	0.20	< .001	-1.92	-1.13
Negative tropical cyclone experiences count	4.61	0.96	< .001	2.72	6.49
Time X Negative tropical cyclone experiences count	-0.30	0.16	.069	-0.62	0.02
Covariates					
Age	-0.03	0.04	.423	-0.10	0.04
Gender (ref = man)					
Woman	2.74	1.00	.006	0.79	4.70
Ethnicity (ref = White, non-Hispanic)					
Black, non-Hispanic	1.28	1.71	.456	-2.08	4.63
Other, non-Hispanic	-2.48	2.01	.217	-6.43	1.46
Hispanic	1.32	1.39	.342	-1.40	4.03
2+, non-Hispanic	0.39	1.95	.844	-3.44	4.21
Household income	-0.17	0.12	.161	-0.41	0.07
Education (ref = no college degree)					
College degree	-2.88	1.00	.004	-4.85	-0.92
State (ref = Texas)					
Florida	3.14	1.06	.003	1.07	5.22
Intercept	16.55	2.81	< .001	11.05	22.06
Random effects parameters					
Participant					
Negative tropical cyclone experiences variance	6.42	6.10		1.00	41.27
Intercept variance	94.74	17.84		65.49	137.04
Negative tropical cyclone experiences - intercept covariance	-2.33	14.55		-30.85	26.19
Residual variance	175.01	12.45		152.23	201.20
Model fit	$\chi^2(12) = 175.89$, $p < .001$, AIC = 45453.64, BIC = 45543.69				

Linear mixed effects model predicting personal tropical cyclone risk perceptions (%) from the interaction between time and self-reported negative tropical cyclone experiences total count (cumulative up to Wave 3), controlling for covariates. *Note.* CI = confidence intervals; LL = lower limit; UL = upper limit; Ref = reference comparison group; AIC = Akaike's Information Criterion; BIC = Bayesian Information Criterion; Personal tropical cyclone risk perceptions (home damage and injury composite) ranged from *0%* to *100%*; Models were estimated using Wave 5 poststratification survey weights, maximum likelihood estimation, and an unstructured covariance matrix.

Table S17.

Variable	Coefficient	SE_{Robust}	p	95% CI LL	95% CI UL
Main predictors					
Time	-0.05	0.05	.345	-0.15	0.05
Adaptation	0.05	0.03	.118	-0.01	0.12
Covariates					
Age	-0.01	0.004	.051	-0.02	< 0.001
Gender (ref = man)					
Woman	-0.15	0.15	.327	-0.44	0.15
Ethnicity (ref = White, non-Hispanic)					
Black, non-Hispanic	0.65	0.22	.003	0.22	1.09
Other, non-Hispanic	0.74	0.38	.048	0.01	1.48
Hispanic	0.47	0.19	.015	0.09	0.85
2+, non-Hispanic	0.10	0.46	.829	-0.81	1.01
Household income	-0.01	0.02	.485	-0.05	0.02
Education (ref = no college degree)					
College degree	0.33	0.15	.027	0.04	0.62
State (ref = Texas)					
Florida	-0.11	0.16	.483	-0.42	0.20
Cut 1	-0.11	0.45		-1.00	0.78
Cut 2	0.64	0.46		-0.25	1.54
Random effects parameters					
Participant					
Adaptation variance	0.06	0.04		0.02	0.24
Intercept variance	3.02	0.96		1.62	5.65
Adaptation - intercept covariance	-0.32	0.19	.094	-0.70	0.55
Model fit	$\chi^2(11) = 30.87$, $p = .001$, AIC = 4776.41, BIC = 4860.73				

Ordered probit mixed effects model predicting future tropical cyclone risk perceptions (0 = *less or same intensity and frequency*, 1 = *more intense or frequent*, 2 = *more intense and frequent*) from time, cumulative self-reported tropical cyclone household adaptation count, and covariates (main effects). *Note.* CI = confidence intervals, LL = lower limit; UL = upper limit; Ref = reference comparison group; AIC = Akaike's Information Criterion; BIC = Bayesian Information Criterion; Wave 5 poststratification survey weights and an unstructured covariance matrix were used.

Table S18.

Variable	Coefficient	SE_{Robust}	*p*	95% CI LL	95% CI UL
Main predictors					
Time	0.01	0.09	.938	-0.17	0.18
Adaptation	0.13	0.10	.198	-0.07	0.32
Time X Adaptation	-0.02	0.02	.430	-0.06	0.03
Covariates					
Age	-0.01	0.00 4	.050	-0.02	< 0.001
Gender (ref = man)					
Woman	-0.14	0.15	.341	-0.44	0.15
Ethnicity (ref = White, non-Hispanic)					
Black, non-Hispanic	0.66	0.22	.003	0.22	1.10
Other, non-Hispanic	0.75	0.38	.049	0.003	1.49
Hispanic	0.48	0.19	.014	0.10	0.86
2+, non-Hispanic	0.10	0.46	.832	-0.81	1.01
Household income	-0.01	0.02	.496	-0.05	0.02
Education (ref = no college degree)					
College degree	0.33	0.15	.028	0.04	0.63
State (ref =Texas)					
Florida	-0.11	0.16	.488	-0.42	0.20
Cut 1	0.12	0.53		-0.93	1.16
Cut 2	0.87	0.54		-0.20	1.94
Random effects parameters					
Participant					
Adaptation variance	0.06	0.04		0.02	0.24
Intercept variance	3.04	0.97		1.63	5.68
Adaptation - intercept covariance	-0.33	0.19	.093	-0.71	0.54
Model fit	$\chi^2(12) = 30.64$, $p = .002$, AIC = 4776.43, BIC = 4866.02				

Ordered probit mixed effects model predicting future tropical cyclone risk perceptions (0 = *less or same intensity and frequency*, 1 = *more intense or frequent*, 2 = *more intense and frequent*) from time, cumulative self-reported tropical cyclone household adaptation count, time X adaptation count interaction, and covariates. *Note.* CI = confidence intervals, LL = lower limit; UL = upper limit; Ref = reference comparison group; AIC = Akaike's Information Criterion; BIC = Bayesian Information Criterion; Wave 5 poststratification survey weights and an unstructured covariance matrix were used.

Table S19.

Variable	Coefficient	SE_{Robust}	p	95% CI LL	95% CI UL
Main predictors					
Time	-0.03	0.05	.498	-0.12	0.06
Negative tropical cyclone experiences count	0.09	0.08	.280	-0.07	0.25
Covariates					
Age	-0.01	0.004	.053	-0.02	< 0.001
Gender (ref = man)					
Woman	-0.19	0.14	.176	-0.48	0.09
Ethnicity (ref = White, non-Hispanic)					
Black, non-Hispanic	0.53	0.25	.033	0.05	1.01
Other, non-Hispanic	0.72	0.36	.045	0.02	1.42
Hispanic	0.50	0.19	.008	0.13	0.86
2+, non-Hispanic	0.04	0.44	.921	-0.81	0.90
Household income	-0.01	0.02	.553	-0.04	0.02
Education (ref = no college degree)					
College degree	0.24	0.16	.133	-0.07	0.55
State (ref = Texas)					
Florida	-0.05	0.15	.722	-0.34	0.23
Cut 1	-0.16	0.43		-1.01	0.69
Cut 2	0.57	0.43		-0.28	1.41
Random effects parameters					
Participant					
Negative tropical cyclone experiences variance	0.30	0.12		0.13	0.67
Intercept variance	2.04	0.47		1.30	3.22
Negative tropical cyclone experiences - intercept covariance	-0.63	0.26	.014	-1.13	-0.13
Model fit	$\chi^2(11) = 26.06$, $p = .006$, AIC = 4860.44, BIC = 4945.20				

Ordered probit mixed effects model predicting future tropical cyclone risk perceptions (0 = *less or same intensity and frequency*, 1 = *more intense or frequent*, 2 = *more intense and frequent*) from time, cumulative self-reported negative tropical cyclone experiences sum count (Wave 3), and covariates (main effects). *Note.* CI = confidence intervals, LL = lower limit; UL = upper limit; Ref = reference comparison group; AIC = Akaike's Information Criterion; BIC = Bayesian Information Criterion; Wave 5 poststratification survey weights and an unstructured covariance matrix were used.

Table S20.

Variable	Coefficient	SE_{Robust}	*p*	95% CI LL	95% CI UL
Main predictors					
Time	-0.04	0.05	.400	-0.14	0.06
Negative tropical cyclone experiences count	0.02	0.17	.881	-0.30	0.35
Time X Negative tropical cyclone experiences count	0.02	0.03	.644	-0.05	0.08
Covariates					
Age	-0.01	0.004	.054	-0.02	< 0.001
Gender (ref = man)					
Women	-0.19	0.14	.178	-0.48	0.09
Ethnicity (ref = White, non-Hispanic)					
Black, non-Hispanic	0.53	0.24	.030	0.05	1.01
Other, non-Hispanic	0.71	0.36	.046	0.01	1.42
Hispanic	0.50	0.19	.008	0.13	0.86
2+, non-Hispanic	0.05	0.44	.918	-0.81	0.90
Household income	-0.01	0.02	.540	-0.04	0.02
Education (ref = no college degree)					
College degree	0.24	0.16	.123	-0.07	0.55
State (ref = Texas)					
Florida	0.05	0.15	.725	-0.23	0.34
Cut 1	-0.15	0.43		-1.00	0.70
Cut 2	-0.58	0.43		-0.27	1.42
Random effects parameters					
Participant					
Negative tropical cyclone experiences variance	0.30	0.12		0.13	0.67
Intercept variance	2.04	0.47		1.30	3.22
Negative tropical cyclone experiences - intercept covariance	-0.63	0.26	.014	-1.13	-0.13
Model fit	$\chi^2(12) = 26.97$, *p* = .001, AIC = 4861.93, BIC = 4951.98				

Ordered probit mixed effects model predicting future tropical cyclone risk perceptions (0 = *less or same intensity and frequency*, 1 = *more intense or frequent*, 2 = *more intense and frequent*) from time, cumulative self-reported negative tropical cyclone experiences sum count (Wave 3), time X negative tropical cyclone experiences count interaction, and covariates. *Note.* CI = confidence intervals, LL = lower limit; UL = upper limit; Ref = reference comparison group; AIC = Akaike's Information Criterion; BIC = Bayesian Information Criterion; Wave 5 poststratification survey weights and an unstructured covariance matrix were used.
